# Artificial intelligence for Traditional Chinese Medicine classic formula development: a technology readiness assessment using metabolic syndrome as a case study

**DOI:** 10.3389/fphar.2026.1970742

**Published:** 2026-10-28

**Authors:** Zihao Huang, Zhenhong Jiang, Shaowen Hu, Ning Ye, Xingteng Liu, Xinyi Zhan, Zhenhui Wu, Hao Chen, Zihao Zhao, Xinyu Li, Zhenzhong Zang, Chuanyun Xiao, Xiangmeng Qu, Langxin Huang, Haowei Peng, Xiang Zhou, Ming Yang, Huanhua Xu

**Affiliations:** 1 State Key Laboratory for the Modernization of Classical and Famous Prescriptions of Chinese Medicine, Nanchang, China; 2 Jiangxi Key Laboratory of Molecular Medicine, The Second Affiliated Hospital of Nanchang University, Nanchang, China; 3 Jiangxi Science and Technology Infrastructure Center, Jiangxi Computing Center, Nanchang, China; 4 The Affiliated Hospital of Jiangxi University of Chinese Medicine, Nanchang, China; 5 Key Laboratory of Sensing Technology and Biomedical Instruments of Guangdong Province, School of Biomedical Engineering, Shenzhen Campus of Sun Yat-Sen University, Shenzhen, China

**Keywords:** artificial intelligence, knowledge graph (KG), large language model (LLM), metabolic syndrome, network pharmacology, Traditional Chinese Medicine

## Abstract

Traditional Chinese medicine (TCM) formulas, developed through millennia of clinical practice, represent a rich legacy of approximately 100,000 documented prescriptions (99,584 named formula entries in the second edition of the Dictionary of Traditional Chinese Medicine Formulas), yet on average fewer than ten new TCM products were approved annually by China’s NMPA from 2013 to 2024 (77 approvals in total). The experience-based screening paradigm is misaligned with the precision medicine framework demanded by metabolic syndrome (MetS), a multi-target disorder affecting an estimated 1.54 billion adults (95% CrI 1.35–1.76) in 2023. Although artificial intelligence (AI)—encompassing knowledge graphs (KGs), large language models (LLMs), and high-throughput experimentation—has been proposed as a solution, its actual readiness for TCM development remains unclear. This review critically examines KG, LLM, and high-throughput validation technologies enabling precision phytomedicine for MetS, providing a realistic roadmap for AI-driven TCM research and development. The three technology streams exhibit asymmetric readiness. At the stream level, knowledge-graph approaches span Technology Readiness Level (TRL) 3–6, large language model approaches span TRL 1–5, and high-throughput validation platforms span TRL 3–5. LLM development is limited by three TCM-specific hallucination types that lack evaluation benchmarks. KG entity recognition is the most mature component, yet reasoning is hindered by the absence of negative data, missing dose–time dimensions, and unencoded monarch–minister–assistant–envoy hierarchies. High-throughput platforms are technically feasible but remain mismatched to MetS functional endpoints—tumor-derived organoid paradigms show limited direct transferability and would require substantial adaptation. Precision phytomedicine must follow a “foundations before algorithms” sequence: institutionalize negative data, build TCM-Hallu benchmarks, establish functional-endpoint evaluation, and develop AI-assisted regulatory review. Algorithmic advances will remain in an incomplete information space until knowledge infrastructure gaps are closed.

## Graphical Abstract

**Figure:**
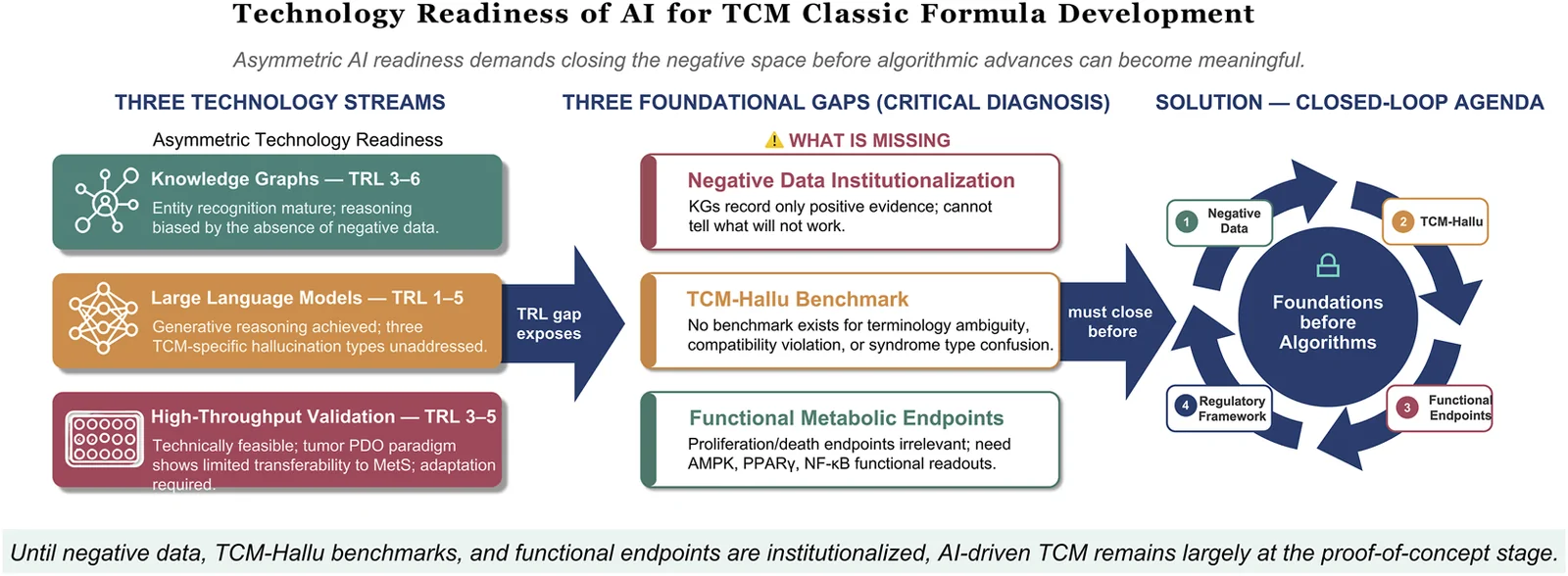

## 1 Introduction

### 1.1 Historical legacy and modern translational challenges of Traditional Chinese Medicine

TCM formulas represent a vast repository of empirical therapeutic knowledge accumulated over millennia. The Huangdi Neijing establishes the classical prescription canon (Unschuld, 2003; He et al., 2017); classic formulas such as Huangqi (*Astragalus mongholicus* Bunge (Fabaceae; root; syn. *A. membranaceus* (Fisch.) Bunge))–Danshen (*Salvia miltiorrhiza* Bunge (Lamiaceae; root and rhizome))–Gegen (*Pueraria lobata* (Willd.) Ohwi (Fabaceae; root)) have demonstrated multi-pathway therapeutic potential in modern pharmacological studies (Liang H. et al., 2025; Xu et al., 2022). Yet the number of TCM formulas transformed into approved modern drugs remains strikingly low—on average fewer than 10 per year from 2013 to 2024 (77 NMPA approvals) (Long et al., 2025)—despite the existence of approximately 100,000 documented formulas (99,584 named entries in the second edition of the Dictionary of Traditional Chinese Medicine Formulas, counting synonym and modified-form entries without deduplication) (Peng, 2019), a translational gap that has prompted leading experts to call for systemic reform in TCM research and development (Zhang B. et al., 2025).

This bottleneck is not primarily pharmacological; it is epistemological (Jiang et al., 2025). Three structural constraints underlie this stagnation: (1) knowledge fragmentation—the therapeutic rationale of classic formulas is distributed across classical texts, modern clinical records, and pharmacological studies without unified semantic representation; (2) inference opacity—the multi-target, network-level mechanisms of formulas resist reductionist single-target explanatory models; and (3) validation inefficiency—the cost and speed of traditional experimental validation cannot keep pace with the combinatorial complexity of formula optimization (Li et al., 2025a; Hopkins, 2007). Through this lens, we adopt a Chinese medicine modernization perspective to evaluate how AI can faithfully encode, reason about, and revitalize the indigenous medical knowledge embedded in classical TCM formulas.

### 1.2 Metabolic syndrome as a translational testbed

Metabolic syndrome (MetS) is a cluster of interconnected physiological abnormalities—including central obesity, dyslipidemia, hypertension, and hyperglycemia (Engin, 2024; Cornier et al., 2008; O’Neill and O’Driscoll, 2015)—that represents an ideal translational testbed (Zhang J. et al., 2026) for TCM research. With an estimated 1.54 billion affected adults worldwide (95% CrI 1.35–1.76) in 2023 (Noubiap et al., 2025), the multi-target pathology of MetS aligns fundamentally with the multi-component, multi-pathway therapeutic approach of TCM. A single-target intervention cannot adequately address MetS (Proschak et al., 2019); the disease demands precisely the holistic, multi-pathway modulation that TCM formulas are theorized to provide (Pathak et al., 2025a; Gu et al., 2025).

This alignment creates both an opportunity and a diagnostic constraint. By using MetS as a consistent case study throughout this review—rather than treating it as a peripheral illustration—we systematically examine whether AI technologies are genuinely ready to support TCM drug development for complex metabolic diseases, or whether current capabilities remain limited to proof-of-concept demonstrations (Valdez Vega et al., 2025; Zhang et al., 2023).

### 1.3 Existing reviews and positioning of this work

Several recent reviews have examined AI applications in TCM (Han X. et al., 2026; Bhadra et al., 2025). However, three critical gaps persist in the existing literature. First, prior reviews treat MetS as an occasional example rather than a structured translational case (Chen Z. et al., 2025; Lu et al., 2024); consequently, the disease-specific constraints on validation platform design are rarely examined. Second, no existing review has applied a structured technology readiness assessment to the three key AI technology streams relevant to TCM development (Salvador-Carulla et al., 2024). Third, the three TCM-specific hallucination categories unique to LLM-generated TCM content have not been systematically classified (Basu and Huynh, 2026; Li Y. et al., 2025).

This review makes three distinctive contributions: (1) MetS serves as a threading translational case applied consistently across all sections (Noubiap et al., 2025; Neeland et al., 2024); (2) technology readiness levels (TRL) provide a structured, cross-domain assessment framework for comparing KG, LLM, and high-throughput experimentation (O’Connell et al., 2024); and (3) three TCM-specific hallucination categories—terminology ambiguity, compatibility-rule violation, and syndrome-cross-type confusion—are systematically identified and assessed (Basu and Huynh, 2026; Sun et al., 2025).

### 1.4 A roadmap for this review

This review is organized into five content sections (Sections 3–7) addressing the three technological pillars and their integration. Section 3 (Knowledge Graphs) addresses the knowledge fragmentation challenge, reviewing TCM-KG construction methods, platform comparisons, and three critical structural gaps—the absence of negative data, missing dose–time dimensions, and unencoded monarch–minister–assistant–envoy hierarchies. Section 4 (Large Language Models) addresses the inference opacity challenge, analyzing TCM-specific LLM pathways and three hallucination categories unique to TCM content. Section 5 (Formula Screening) examines synergies between KG and LLM in formula recommendation, introducing multi-agent architectures and TRL grading for the integrated system. Section 6 (High-Throughput Validation) addresses validation inefficiency, reviewing organoids, organ-on-chip systems, and AI-HTS, with the MetS–tumor PDO mismatch exposing systematic constraints in validation platform design. Section 7 synthesizes bottlenecks across all four areas and proposes a prioritized research agenda. The overall framework, associated bottleneck mapping, and priority research agenda are depicted in Figure 1. Sections 6.5 and 6.6 assess the pharmacological and clinical evidence base for MetS-relevant formulas.

**FIGURE 1:**
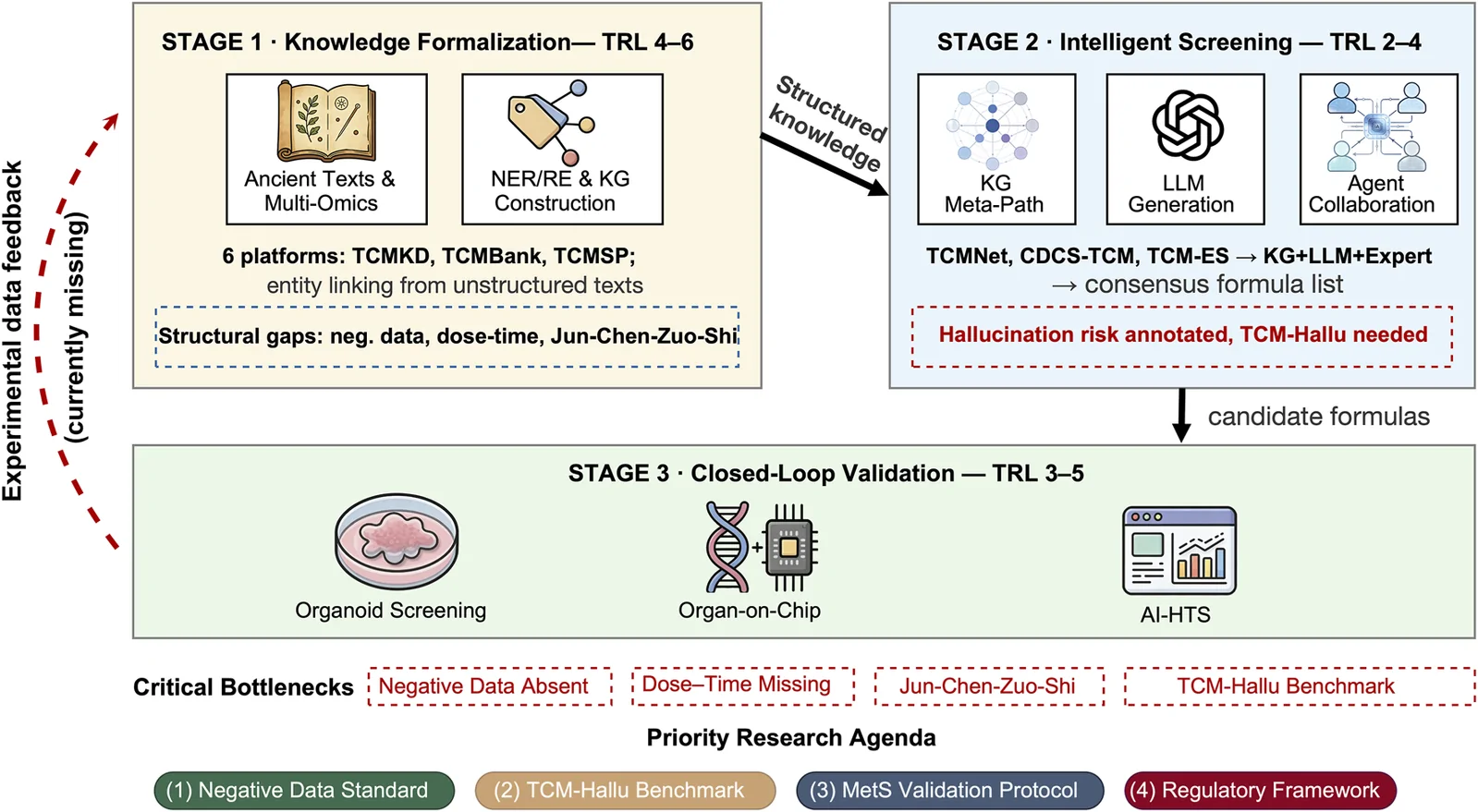
Three-stage framework for the AI-enabled precision phytomedicine for classic formula R&D, with bottleneck mapping and priority research agenda.

It is essential to emphasize that these three technology streams are not parallel pillars—they form what we propose as a quasi-closed loop: KG provides structured knowledge representation, LLM enables cross-entity reasoning on that knowledge base, and high-throughput experiments supply validation feedback that should theoretically be reinjected into the knowledge base. The loop is termed quasi-closed rather than fully closed because the “experimental validation to knowledge reinjection” leg currently lacks mature engineering instances (see Sections 7.1 and 7.2). A break at any leg of this quasi-closed loop will cause the precision phytomedicine translation to stall at the proof-of-concept stage.

It is important to distinguish this work from that of Hong et al. (Hong et al., 2026), who proposed a multi-scale target analysis framework for AI-driven TCM from the perspective of material hierarchy (single compound to herb to formula) and introduced the concept of a computational–experimental closed loop. The present review approaches the same problem from the perspective of technology readiness asymmetry: using MetS as a disease-specific case study, systematically mapping structural gaps across three technology streams, and identifying a three-category TCM-specific hallucination taxonomy. The two papers are complementary rather than overlapping—Hong et al. provide a constructive framework (Hong et al., 2026), whereas this review delivers a critical diagnostic assessment.

## 2 Search strategy

### 2.1 Review approach and search strategy

This article is a critical narrative review rather than a screened systematic review: the methodological heterogeneity of knowledge-graph construction, LLM fine-tuning, organoid platforms and clinical trial designs precludes pooled effect-size synthesis; a PRISMA flow diagram is therefore not applicable; and rigid inclusion criteria would exclude precisely the early proof-of-concept work this review needs to examine. We therefore combined a structured database search with citation tracing of key reviews. Four databases (PubMed, Web of Science, CNKI and Wanfang) were searched for records published between January 2007 and June 2026, the window opening with the introduction of network pharmacology (Hopkins, 2007). The full Boolean strings, record counts and execution dates for all four databases are provided in Supplementary Table S3; in PubMed, the primary strategy combining AI-technology and TCM-concept terms retrieved 1,221 records, and 77 after adding metabolic-syndrome terms (retrieved 29 September 2026). Records were selected for relevance to the three technology streams and the MetS case study, and the 149 cited works constitute the final evidence base.

### 2.2 Technology readiness assessment methodology

To compare heterogeneous technologies on a common scale while avoiding the conflation of distinct maturity dimensions noted in the editorial assessment, we define a readiness-assessment scheme adapted from the TRL-IS framework (Salvador-Carulla et al., 2024). Three design choices distinguish it from a single ordinal rating. TRL remains a descriptive reference tool rather than a standardized certification instrument: it provides a cross-domain comparison benchmark, not a prediction of industrialization timelines.

### 2.3 First, rating unit

Every rating is assigned to one of three units: (i) technology stream (knowledge graphs, large language models, high-throughput validation), reported in the Abstract and Conclusions only; (ii) component (e.g., TCM named-entity recognition, GNN graph reasoning, LLM formula recommendation, multi-agent orchestration, GraphRAG, metabolic organoid validation, organ-chip integration, AI-HTS screening), reported in Table 2 and Section 5.3; and (iii) stage (knowledge formalization, intelligent screening, closed-loop validation), reported in Figure 1. A stream-level rating is the range spanned by its components and is never presented as a single component’s value.

### 2.4 Second, intended use

Readiness is intended-use dependent: each component rating specifies the use being scored—retrieval (extracting known associations), hypothesis generation (proposing candidate relationships), candidate prioritization (ranking among alternatives), or regulatory evidence (supporting preclinical/clinical decisions). A component may carry different ratings for different uses; we report the use with the strongest evidence base and flag others.

### 2.5 Third, full-scale definitions

We define TRL 1–9, with parallel wording for software-type and assay-type components (Supplementary Table S1): TRL 1, basic principles observed; TRL 2, technology concept formulated for the TCM/MetS context; TRL 3, experimental proof of concept on a public benchmark or prototype dataset; TRL 4, laboratory validation (component validated *in vitro* or on domain corpora with reported metrics); TRL 5, relevant-environment validation (*in vivo* models, or deployment on real TCM text or knowledge graphs at research scale); TRL 6, engineering validation (integrated pipeline, preclinical GMP-grade or production-grade system); TRL 7, system demonstration in an operational environment; TRL 8, system completed and qualified; TRL 9, system proven in deployment. Levels 7–9 are not assigned in this review and are stated as such.

Beyond the ordinal level, each component is scored on three explicit criteria: prospective utility (evidence of predicting previously unknown, subsequently validated results versus retrospective recovery of known associations), independent replication (results reproduced by a group other than the original developers), and non-AI baseline comparison (performance reported against a non-ML baseline where applicable). These scores, the supporting evidence per criterion, and the decision rule applied are tabulated in Supplementary Table S1. Retrospective-recovery performance (e.g., P@30 on curated benchmark sets) is explicitly not treated as evidence of prospective utility.

### 2.6 Rating process

Ratings were assigned by the author team through consensus assessment against the published criteria described above: each component rating was drafted from the cited primary evidence, reviewed by all authors, and finalized only once no unresolved objection remained. Because this review does not claim inter-rater reliability testing, no agreement statistic is reported; the full rating evidence underlying every score, including the criterion-to-literature mapping, is provided in Supplementary Table S1 to allow independent verification of each rating.

Two modes of AI contribution are distinguished throughout this review: retrospective mining, which recovers known associations from literature and case records, and prospective generation, which predicts outcomes that have not yet been experimentally verified. The TRL ratings in Section 5.3 accordingly consider three explicit dimensions for each technology: prospective utility, independent replication, and comparison against non-AI baselines.

## 3 Knowledge graphs for classic Chinese medicine

### 3.1 Principles and current state of TCM-KG construction

Transforming TCM knowledge into a computationally tractable form requires first converting formula–herb–target–syndrome information scattered across classical texts and modern literature into machine-processable entity–relationship triples (Xiang et al., 2025; Qu et al., 2024). NER/RE methods in the TCM domain have advanced through four stages: rule-based methods, statistical machine-learning methods, deep-learning-based methods, and pre-training-based methods (Xiong, 2025; Qu et al., 2020). The primary bottleneck in TCM text processing is not algorithmic accuracy but long-range semantic associations—the compatibility logic of jun-chen-zuo-shi (monarch–minister–assistant–envoy) frequently spans multiple sentences, and earlier methods (dictionary matching, CRF, BiLSTM-CRF) were constrained by sentence boundaries. The document-level graph convolutional network developed by Xu et al. represents the first departure from this limitation (Xu W. et al., 2024): it simultaneously extracts entities and relations across entire medical cases, capturing cross-sentence syndrome differentiation logic through graph structures. This approach has not yet been validated on MetS medical cases, and no public evaluation benchmark is currently available (Yea et al., 2025; Zhao et al., 2015).

### 3.2 TCM-KG platforms: a comparative landscape

At the platform level, several representative TCM-KG systems have been developed. TCMSP catalogs 499 medicinal materials and 29,384 compounds (Ru et al., 2014), establishing foundational drug–target and drug–disease associations. TCMID encompasses over 47,000 formulas (Xue et al., 2012), providing ternary mappings of medicinal material–compound–target. HIT includes over 1,300 medicinal materials and 5,860 targets (Ye et al., 2011). SymMap aligns 1,717 TCM symptoms with their modern medical counterparts (Wu et al., 2019). TCMKD contains seven entity types and 2,324 nodes (Xiao W. et al., 2025), with network visualization capabilities. TCMBank hosts 9,162 medicinal materials and 61,966 compounds (Lv et al., 2023). A horizontal comparison of these six platforms is presented in Table 1.

**TABLE 1:** Horizontal comparison of representative TCM-KG systems.

Platform	Entities & scale	Relationship types	MetS coverage	API Availability	References/Year
TCMSP	499 herbs, 29,384 compounds	Drug-target, drug-disease	Low (no MetS-specific entities)	Web only, no API	(Ru et al., 2014) 2014
TCMID	47,000+ formulas	Herb-compound-target	Low to moderate	Limited API	(Xue et al., 2012) 2013
HIT	1,300+ herbs, 5,860 targets	Herb-target, compound-target	Low	Web query only	(Ye et al., 2011) 2011
SymMap	1,717 TCM symptoms	Symptom-herb, herb-modern symptom	Low (generic TCM, no MetS filter)	REST API available	(Wu et al., 2019) 2019
TCMKD	7 entity types, 2,324 nodes	7 relationship types	Low to moderate (self-assessed)	Web + SPARQL	(Xiao et al., 2025a) 2024
TCMBank	9,162 herbs, 61,966 compounds	Herb-compound-target-disease	Low	Limited API	(Lv et al., 2023) 2023

These platforms build upon a growing body of methodological and review literature on TCM knowledge-graph construction and AI integration (Zhao et al., 2023; Zhou et al., 2024). Disease-specific KGs targeting particular conditions—such as the diabetic retinopathy diagnosis and treatment KG by Xiao et al. (Xiao L. et al., 2025)—represent targeted applications. Common shortcomings persist across existing platforms: all adopt a general-purpose architecture without MetS-specific design, and API openness and interoperability remain insufficient for seamless integration with LLM reasoning pipelines. A quantitative assessment using ten primary MetS syndrome patterns reported in two real-world cohorts (Axinbai et al., 2024; Xia et al., 2020) as the reference set shows that the mean coverage of MetS-related syndrome entities across the six platforms is below 30% (20.0% with documented synonyms included and 15.0% under exact-name matching). Of these, SymMap and TCMKD provide syndrome layers (233 and 262 entries, respectively), whereas TCMSP, TCMBank, HIT and TCMID expose no syndrome entity class in their published data scopes; the interfaces of HIT and TCMID were additionally unreachable at the time of assessment. Per-pattern results and the query rules are provided in Supplementary Table S2. Consequently, the fundamental question of which formulas modulate MetS-related targets cannot be answered by existing architectures—a consequence of product positioning rather than a technical deficiency (Perdomo-Quinteiro and Belmonte-Hernández, 2024; Zhang and Hao, 2024). The overall pipeline from multi-source data integration to ranked formula candidate output is shown in Figure 2.

**FIGURE 2:**
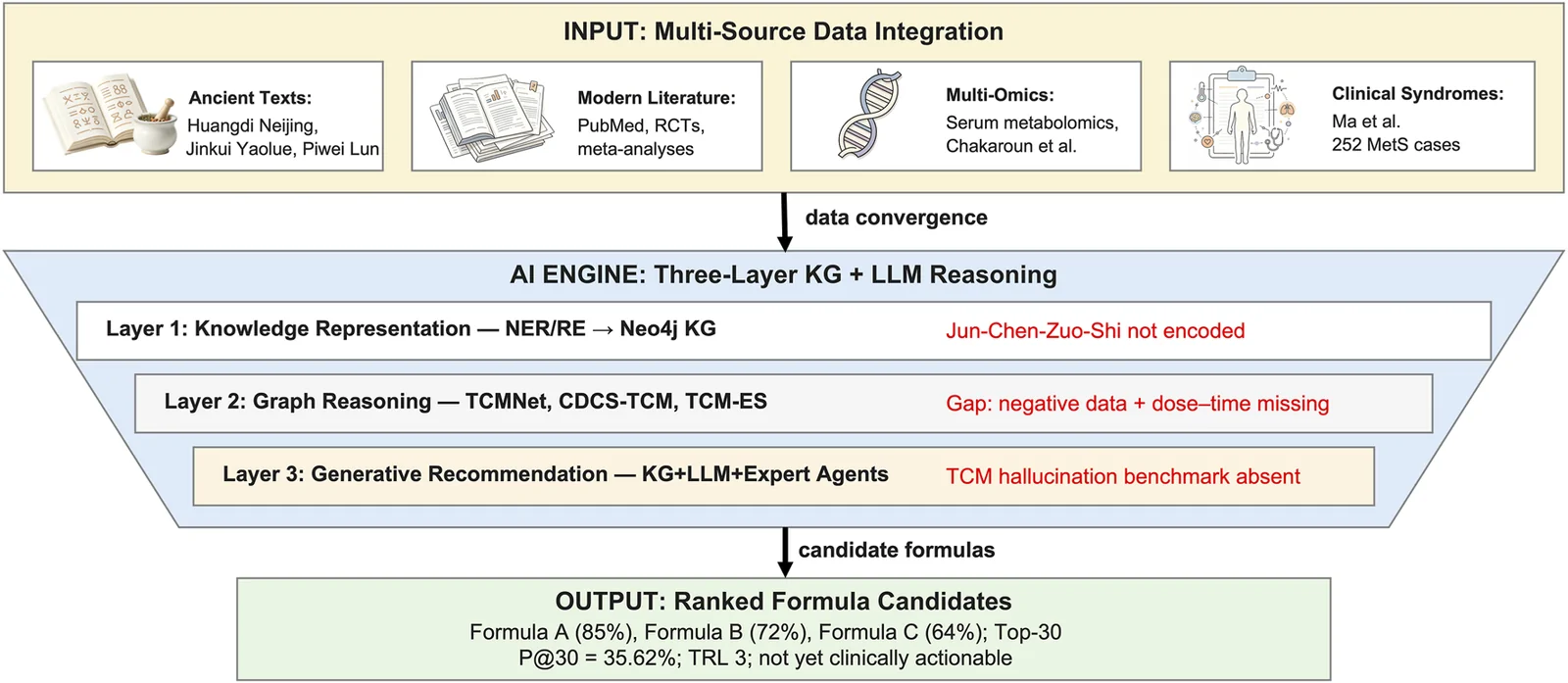
Three-layer pipeline of the AI-enabled precision phytomedicine framework (input standardization, mechanism reasoning, output translation). The output layer remains at TRL 3 (*in silico* proof of concept) and is not yet clinically actionable.

### 3.3 Three structural gaps in TCM-KG reasoning

Gap 1: Absence of Negative Data. The dominant mode in TCM-KG construction is positive-evidence accumulation—adding confirmed herb–target (Zeng et al., 2024), formula–efficacy, and syndrome–treatment relationships. Negative data—confirmed absent effects, failed clinical observations, and incompatible combinations—are systematically absent. This creates a reasoning bias: the KG can indicate what may work but cannot indicate what will not work. For drug development, the absence of negative data is not a minor gap; it is a fundamental constraint on the reliability of any KG-supported recommendation (Montoya et al., 2025; De La Fuente et al., 2025).

Gap 2: Missing Dose–Response and Temporal Dimensions. TCM-KG edges represent directional relationships (“herb A treats symptom B”) but rarely encode dose–response curves or temporal dynamics. The therapeutic effect of most TCM formulas is highly dose-dependent and time-dependent; a KG that records “Danshen treats coronary heart disease” without encoding the dose range and treatment duration is pharmacologically uninformative (Gutierrez et al., 2024; Nogales et al., 2022).

Gap 3: Monarch–Minister–Assistant–Envoy Hierarchies Not Encoded. The classical pharmaceutical theory of jun-chen-zuo-shi (monarch–minister–assistant–envoy) organizes herbs within formulas by functional role. Current TCM-KGs encode this hierarchy inconsistently across platforms, rarely in quantified form, and almost never with experimental validation. This omission loses a critical dimension of formula rationale—the same herb plays different roles at different positions within a formula, and this positional context determines its contribution to the overall therapeutic effect (Wu et al., 2025). Decoding and validating such positional contributions is feasible in principle: Wang et al. decoded and experimentally validated the combination principles of Huanglian Jiedu Decoction from a multi-scale perspective (Wang et al., 2020); validated encodings of this kind, however, remain isolated case studies and have not been absorbed into knowledge graph schemas. A platform-level audit (Supplementary Table S2) bears these three observations out directly: the schemas of TCMSP and TCMBank contain no negative-data, dose–response, temporal, or jun-chen-zuo-shi entity or edge attributes, and the two platforms providing syndrome layers cover the ten reference MetS syndrome patterns only partially (SymMap five of ten, 50%; TCMKD seven of ten, 70% under synonym matching), leaving the most frequent clinical patterns unrepresented. Figure 3 provides a schematic overview of the complementary KG (bottom-up) and LLM (top-down) architecture.

**FIGURE 3:**
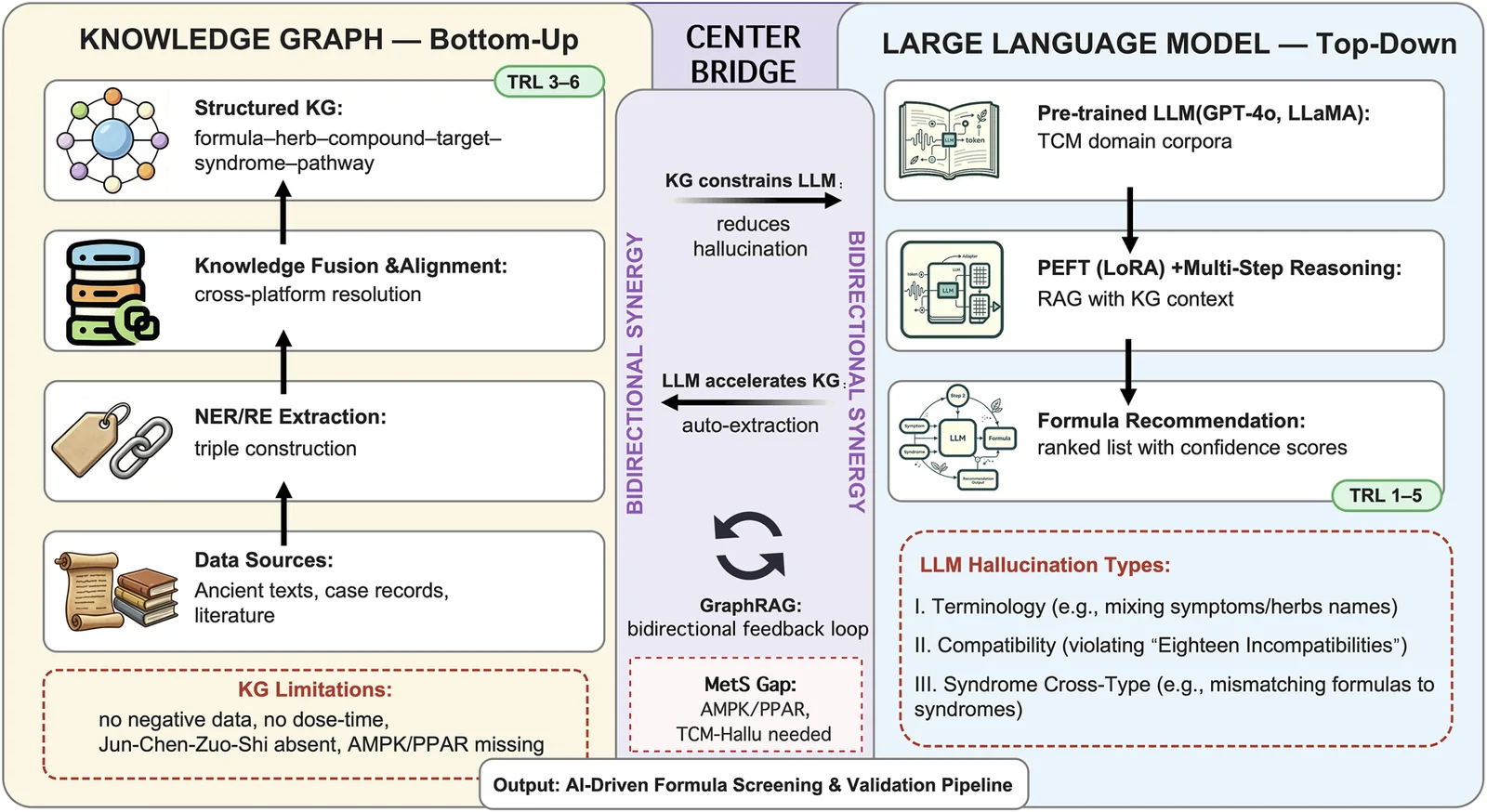
Complementary architecture of knowledge graphs (bottom-up construction) and large language models (top-down reasoning) in TCM knowledge computability. The two technologies provide offsetting rather than overlapping capabilities: knowledge graphs encode verifiable, structured relations, whereas LLMs supply flexible natural-language reasoning over free text; integrated KG–LLM pipelines therefore outperform either component alone.

To remedy these structural gaps at the schema level, we propose that TCM-KG edge attributes should minimally encode six provenance dimensions: (1) species authentication, including the validated botanical name of each constituent herb (verified against World Flora Online and MPNS; Supplementary Table S4); (2) plant part used, since pharmacological activity differs across organs of the same species; (3) processing status (paozhi), because processing alters both efficacy and toxicity profiles; (4) extraction method and dosage form, which determine the phytochemical composition actually delivered; (5) herb proportions within the formula, which carry the jun-chen-zuo-shi hierarchy quantitatively; and (6) the formula text variant from which the record derives, given the documented divergence among classical editions. The platforms compared in Section 3.2 (Table 1) capture none of these dimensions systematically. Without such edge-level provenance, apparently identical herb–target triples aggregated from heterogeneous preparations introduce silent label noise into downstream KG and LLM reasoning.

## 4 Large language models for classic Chinese medicine

### 4.1 TCM-specific LLM technology pathways

Xiao et al. have categorized the core technologies for TCM-specific LLMs into four paradigms (Xiao et al., 2024): retrieval-augmented generation (RAG), mixture-of-experts (MoE), reinforcement learning from human feedback (RLHF), and knowledge distillation—covering the mainstream trajectory from knowledge retrieval augmentation to parameter-efficient fine-tuning. Large model series such as BencaoZhiku represent an important direction in TCM knowledge integration. At the clinical validation level, Hong et al. have reported specific applications of machine learning in TCM quality control and diagnosis (Hong et al., 2025). Current specialized LLMs, however, generally lack publicly disclosed technical details and evaluation data, leaving their clinical reliability without independent verification (Liu et al., 2025; Zhao et al., 2025). Overall, the technical pathway for TCM LLMs has taken initial shape, yet a substantial gap persists between model capability validation and deployment in clinical practice (Han C. et al., 2026; Shataer et al., 2025).

### 4.2 Fine-tuning strategies and domain adaptation

Fine-tuning strategy determines the extent to which general-purpose LLMs can be adapted to the TCM domain (Bonfigli et al., 2024). Current approaches fall into two principal categories: parameter-efficient fine-tuning (e.g., LoRA, adapter layers) and continued domain pre-training (Han C. et al., 2026). Tong et al.'s XuanHuGPT achieved TCM domain adaptation through parameter-efficient fine-tuning (Tong et al., 2025), while Li et al.'s TCM-DS explored LLM-based recommendation of medicine–food homologous formulas (Li X. et al., 2025). In terms of knowledge enhancement, the MedKA framework proposed by Deng et al. improves the factual accuracy of medical LLMs through KG-augmented methods (Deng et al., 2025). This approach aligns with the KG–LLM dual-engine paradigm discussed in Section 5.1, in which the KG serves not merely as a passive knowledge repository but also provides real-time structural constraints during LLM generation (Luo et al., 2024). The performance boundaries of fine-tuning strategies in TCM contexts have yet to be systematically compared; the extent to which different fine-tuning methods can suppress the three TCM-specific hallucination categories remains unquantified (Zhao et al., 2025; Li X. et al., 2025).

### 4.3 TCM-specific hallucination taxonomy

The deployment of LLMs in TCM contexts, particularly for formula recommendation and syndrome differentiation, exposes three hallucination categories not adequately addressed by existing medical LLM benchmarks (Pandit et al., 2025; Kim et al., 2025). We constructed this taxonomy by synthesizing published studies and reviews of LLM performance in TCM-related tasks (Chen C. et al., 2025; Ren et al., 2025; Yuan et al., 2025) with the pharmacological literature on herb compatibility (Xie and Wang, 2023) and our analysis of TCM diagnostic requirements; we propose it as an analytical framework whose validation path is the TCM-Hallu benchmark initiative (Section 7.2). Of the three categories, compatibility-rule violation is specific to TCM, whereas terminology ambiguity and syndrome cross-type confusion have analogues in other specialized domains.

Type I: Terminology Ambiguity. TCM terminology is polysemous and historically layered (Chen C. et al., 2025). A single term such as “qingre” (clearing heat) can refer to different pharmacological mechanisms depending on context—antipyretic, anti-inflammatory, or metabolic regulation. When LLMs process classical TCM texts, the ambiguity of these terms can propagate into generated recommendations, creating therapeutic inconsistencies that are difficult to detect without domain expert review (Ren et al., 2025).

Type II: Compatibility-Rule Violation. TCM pharmacology includes well-documented compatibility rules—certain herb combinations produce toxic effects, reduce efficacy, or generate adverse reactions (Xie and Wang, 2023). LLMs trained primarily on positive-effect literature may generate recommendations that violate these rules without warning, because negative-effect information is underrepresented in the training data (Yuan et al., 2025).

Type III: Syndrome Cross-Type Confusion. TCM syndrome (zheng) differentiation requires distinguishing between syndromes that share clinical manifestations but have different underlying pathomechanisms—a task analogous to the most challenging problems in differential diagnosis (Liu et al., 2025). LLMs may generate recommendations appropriate for one syndrome type but inappropriate for another, with the error propagating into downstream formula recommendations (Zhao et al., 2025). A typology of these three TCM-specific hallucination categories is provided in Figure 4.

**FIGURE 4:**
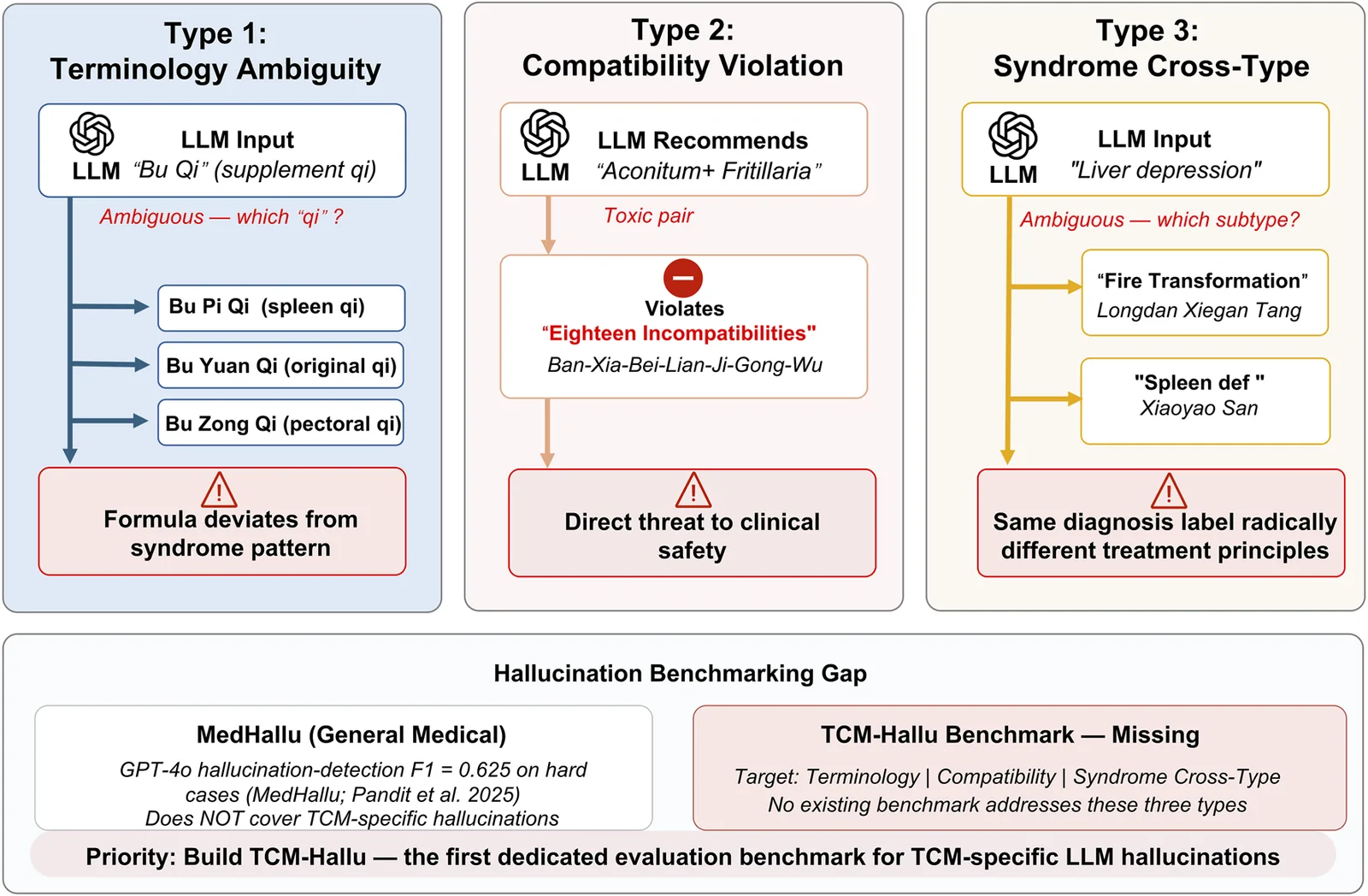
Three types of TCM-specific LLM hallucinations: (Type 1) terminology ambiguity; (Type 2) compatibility-rule violation of the “Eighteen Incompatibilities” rule; (Type 3) syndrome cross-type mismatching.

As discussed in Section 7.2, establishing the proposed TCM-Hallu benchmark—a dedicated hallucination evaluation dataset for TCM LLMs—is a prerequisite for measuring and mitigating these three hallucination categories (Basu and Huynh, 2026). The benchmark should include annotated examples of all three types, evaluated across different model architectures and fine-tuning strategies. Currently, no such benchmark exists; generic medical hallucination benchmarks (e.g., MedHallu) (Pandit et al., 2025) focus on modern biomedical terminology and cannot capture the TCM-specific ambiguity of terms such as “qingre” or the combinatorial logic of compatibility rules (Xu Z. et al., 2024; Asgari et al., 2025). These hallucination patterns pose a direct threat to the fidelity of transmitted traditional medicine knowledge, as erroneous model outputs may corrupt dosage records, conflate distinct botanical identities, or fabricate unsubstantiated therapeutic claims that propagate through downstream applications.

## 5 Formula screening: synergies between KG and LLM

### 5.1 KG-enhanced LLM for formula recommendation

Once a KG has organized formula–herb–target–syndrome relationships into a structured network, LLMs can perform multi-step reasoning on this knowledge foundation (Zhang B. et al., 2026; Zhai et al., 2025). Current KG-driven LLM approaches to formula recommendation proceed along four technical paths. The first path employs semantic weighting: TCMNet incorporates target literature weights into proximity calculations (Tan et al., 2026), yielding statistically significant differences between weighted and unweighted results in Parkinson’s disease cases. These data reflect disease-specific discrimination effects; translation to the MetS context would require retraining on core pathways including AMPK, PPARγ, and NF-κB (Yang et al., 2025). The second path uses dynamic causal modeling: CDCS-TCM tracks temporal changes in causal relationships among substances during metabolic processes using complex network theory (Gao et al., 2024). This method has been validated only for intravenous Xiangdan injection; its extension to orally administered compound formulas and metabolic diseases awaits verification. The third path employs embedding space learning: TCM-ES maps syndrome–formula pairs into continuous vectors, although the semantic ambiguity introduced by vectorizing discrete concepts remains unresolved (Li et al., 2025d). The fourth path pursues *de novo* design: Wang et al. employed RF-QSAR with LSTM-based reinforcement learning targeting the A1/A2A dual receptor (Wang P. et al., 2025), and a separate study used GPT-4o knowledge distillation to achieve a P@30 of 35.62% (Wang X. et al., 2025). Both efforts are limited to single-target molecules and lack constraint mechanisms grounded in TCM compatibility theory (Li J. et al., 2025). The common bottleneck across these approaches is that the upper bound of all reasoning paths is governed by KG coverage: insufficient negative data (Montoya et al., 2025), absent dose–time relationships (Gutierrez et al., 2024), and the scarcity of disease-specific KGs collectively constrain how far KG-driven formula recommendation can advance; even disease-specific pharmacology remains largely outside computable graphs—the AMPK-centered therapeutic evidence in MASLD, for example, is consolidated in narrative reviews (An et al., 2025), a pattern consistent with methodological assessments of data integration in precision medicine (Okyere et al., 2025).

The latest evolution of retrieval-augmented generation—GraphRAG—is particularly well suited to the graph-structured nature of TCM knowledge (Edge et al., 2026). Traditional RAG retrieves flat text chunks on the basis of vector similarity, failing to capture the multi-layer relationships among formulas, herbs, ingredients, targets, pathways, and syndromes (Lewis et al., 2026). GraphRAG traverses the knowledge graph directly during the retrieval phase, enabling generation to follow semantic paths within the knowledge structure. For TCM domains, where knowledge is inherently graph-structured, GraphRAG may constrain hallucinations more effectively than traditional RAG (Luo et al., 2024). The GraphRAG foundation model was described by Edge et al. (Edge et al., 2026); its applications in TCM remain uncharted territory warranting priority exploration (Sha et al., 2025).

### 5.2 Multi-agent architectures for formula design

Multi-agent architectures offer an engineering approach to the complex decision-making demands of formula design. The AgentClinic benchmark established by Schmidgall et al. (Schmidgall et al., 2026) provides a relevant reference: multi-agent systems achieved 60.3% accuracy on standardized MedQA tasks, approaching the level of individual licensed physicians, yet scored only 8.6% on complex clinical decision-making tasks lacking explicit guidelines (Liu Y. et al., 2026)—close to random chance. This finding has direct implications for TCM formula screening: formula recommendation lies precisely between an examination with standard answers and a clinical decision without them. Syndrome classification is anchored by canonical texts, yet the individualized prescription for a specific MetS patient remains heavily dependent on experiential judgment (Zhang J. et al., 2026; Axinbai et al., 2024). Prioritizing knowledge base completeness—for instance, ensuring that the KG covers the Eighteen Incompatibilities, Nineteen Antagonisms, and jun-chen-zuo-shi hierarchies (Wang et al., 2020)—may therefore prove more effective than increasing agent count or iteration rounds. An optimal multi-agent system for MetS formula screening should comprise three coordinated modules (He J. et al., 2026; Loecker et al., 2026): a KG Agent serving as knowledge gatekeeper to ensure that all recommended relationships have literature or database provenance; an LLM Agent generating cross-entity hypotheses in regions beyond KG coverage, such as inferring from existing pharmacological data that modified Bu Zhong Yi Qi Tang may exert insulin-sensitizing effects in qi–yin deficiency type MetS patients (Peng et al., 2026); and an Expert Agent reviewing candidate formulas individually for compatibility contraindications and jun-chen-zuo-shi integrity. Liang et al.'s MCM multi-agent system has made preliminary efforts in this direction (Liang C. et al., 2025), and the three-stage framework of candidate generation, multi-objective optimization, and experimental validation proposed by Pathak et al. (Pathak et al., 2025b) provides a referenceable iterative verification paradigm (Zhao and Hsieh, 2026).

### 5.3 TRL grading for the KG-LLM integrated system

Drawing on the readiness-assessment scheme defined in Section 2, this review assigns component-level TRL ratings, each tied to its intended use, to key components of the KG–LLM integrated system (Table 2; full scoring sheet in Supplementary Table S1). TCM named entity recognition (NER), for the retrieval use, is rated at TRL 4–5: joint entity–relation extraction models achieve NER F1 above 90% on TCM document-level texts (Xu W. et al., 2024), yet no dedicated NER model tailored to MetS clinical text exists. TCMKD and TCMBank are deployed and publicly accessible, but no MetS-specific, syndrome–formula–target integrated KG has been developed. Knowledge graph construction, for the retrieval use, is rated at TRL 5–6. GNN-based graph reasoning, for the candidate-prioritization use, is rated at TRL 3–4: DREAMwalk improves accuracy on general drug–disease pairs by up to 16.8% (Bang et al., 2023), although it has not been validated on TCM formulas (Chen et al., 2026). The MRHormer multi-scale heterogeneous graph transformer (Wu et al., 2026) has shown promise in herb–target interaction prediction but has yet to be tested on MetS-specific targets. LLM-based formula recommendation, for the hypothesis-generation use, is rated at TRL 3: P@30 reaches 35.62% on a retrospective benchmark (Wang X. et al., 2025), a result that does not in itself constitute evidence of prospective utility; the approach lacks clinical validation, and MetS multi-syndrome scenarios remain untested. Multi-agent collaboration is rated at TRL 2–3 (extrapolated from general-medicine evidence): AgentClinic provides a baseline reference (Schmidgall et al., 2026), but no dedicated agent orchestration scheme addresses TCM formula safety. GraphRAG in the TCM domain is at TRL 1–2 (conceptual stage) (Edge et al., 2026). This TRL distribution reveals a notable asymmetry: the component demanding the greatest reliability—formula recommendation—is at the lowest readiness level, and relatively mature entity recognition technology cannot compensate for structural deficiencies at the reasoning layer.

**TABLE 2:** Technology readiness level (TRL) assessment of key technologies in the KG–LLM integrated system.

Technology	TRL (component-level)	Validation evidence	MetS-specific gaps	Rating unit	Intended use	Primary evidence
TCM NER	TRL 4–5	NER F1 90.7% on TCM document-level texts (Xu et al., 2024a)	No domain NER model for MetS clinical text	Component	Retrieval	(Xu et al., 2024a)
KG Construction	TRL 5–6	TCMKD, TCMBank deployed and publicly accessible (Xiao et al., 2025a; Lv et al., 2023)	No MetS-specific syndrome–formula–target integrated KG	Component	Retrieval	(Xiao et al., 2025a; Lv et al., 2023)
GNN Graph Reasoning	TRL 3–4	DREAMwalk accuracy improvement up to 16.8% (Bang et al., 2023); MRHormer inductive prediction (Wu et al., 2026)	Validated only on general drug–disease pairs, not TCM formulas	Component	Candidate prioritization	(Bang et al., 2023; Wu et al., 2026)
LLM Formula Recommendation	TRL 3	P@30 = 35.62% (Wang et al., 2025b); no clinical validation	MetS multi-syndrome scenarios untested	Component	Hypothesis generation	(Wang et al., 2025b)
Multi-Agent Collaboration	TRL 2–3	AgentClinic: MedQA 60.3%, HLE 8.6% (Schmidgall et al., 2026; Liu et al., 2026a)	No dedicated agent orchestration for TCM formula safety	Component	Candidate prioritization	(Schmidgall et al., 2026; Liu et al., 2026a)
Metabolic Organoid Validation	TRL 3–4	Stem-cell-derived liver organoid maturation: multi-week protocols (Liu et al., 2024a)	Functional metabolic endpoint standardisation missing	Component	Regulatory evidence (preclinical)	(Liu et al., 2024a)
Organ-Chip Integration	TRL 3–4	Intestine–liver dual-chip demonstrated (Negoro et al., 2025)	No chip integrating more than three metabolically relevant tissues reported as of June 2026 (authors’ search)	Component	Regulatory evidence (preclinical)	(Negoro et al., 2025)
AI-HTS Screening	TRL 4–5	AI-assisted HTS workflows have been reviewed elsewhere (Liu et al., 2026b)	Compound formula false-positive accumulation unresolved	Component	Candidate prioritization	(Liu et al., 2026b)

## 6 High-throughput experimental validation

### 6.1 Organoid platforms

Organoid technology has emerged as an important tool for validating the efficacy of TCM formulas (Pushpa Ramesan et al., 2026). Wang et al. reviewed recent advances in human-derived organoids as three-dimensional *in vitro* drug discovery platforms (Wang et al., 2026), and Zhou et al. summarized the application of organoids and organ-on-chip systems in drug screening (Zhou et al., 2025). The 3D ECM organoid high-throughput screening platform developed by Xu et al. demonstrated considerable throughput (Xu Z. et al., 2025): over 10,000 uniform domes were produced within 13 days, and 2,802 compounds were screened. This platform has so far been validated only on rare disease models; its performance in metabolic disease organoids awaits independent verification. Singh et al. reported the latest advances in polycystic ovary syndrome (PCOS) organoid modeling (Singh et al., 2026). PCOS, as a reproductive metabolic disorder closely related to MetS, provides a proof of concept for metabolic organoid differentiation protocols, although the standardized assessment of functional metabolic endpoints remains unresolved. Overall, the maturity of organoid technology in the metabolic disease domain lags considerably behind that in oncology: functional maturation of stem-cell-derived hepatic organoids typically requires several weeks of differentiation; current steatosis induction protocols (e.g., free-fatty-acid exposure) add further culture time before metabolic phenotypes stabilize; and throughput is insufficient to support large-scale formula screening (Pushpa Ramesan et al., 2026; Liu S. et al., 2024; Takebe et al., 2013).

### 6.2 Organ-on-chip platforms

Organ-on-chip technology addresses the multi-organ crosstalk that organoid systems cannot recapitulate. TCM formulas are orally administered multi-component preparations whose *in vivo* effects depend on three mutually reinforcing steps: intestinal absorption, hepatic first-pass metabolism, and target tissue distribution. The genome-edited intestine–liver chip developed by Negoro et al. (Negoro et al., 2025) has rendered this tandem evaluation an operable experimental platform. Zhu et al. reviewed the application of hepatic organoid-on-chip systems in steatohepatitis and individualized metabolic difference assessment (Zhu et al., 2026). Zhao et al., in a comprehensive review published in Nature Reviews Bioengineering, systematically examined integration strategies for organoids and organ-on-chip devices (Zhao et al., 2024), providing an engineering pathway reference for multi-tissue metabolic interaction modeling. Current organ-on-chip platforms (Low et al., 2021) face substantial limitations in the MetS context: the multi-tissue interactions involved in MetS (adipose tissue, liver, pancreas, vasculature, and gut microbiome (Kim et al., 2012)) far exceed the integration capacity of present two-organ chips. System complexity increases superlinearly with each additional tissue module, and as of our literature search in June 2026, we found no reported chip system incorporating more than three metabolically relevant tissues. Furthermore, multi-dimensional efficacy assessment lacks unified weighting criteria. The CKM four-axis biomarker framework (metabolism, immune-inflammation, oxidative stress, biological aging) proposed by Li et al. (Li et al., 2026) provides a referenceable evaluation framework in this regard (Tan et al., 2023; Juguilon et al., 2025).

### 6.3 The MetS-organoid paradigm mismatch

The tumor PDO paradigm, which has produced the most mature and widely adopted organoid validation protocols, is built on three convergent premises: (1) proliferation or death as the primary readout endpoint; (2) single-tissue organoid models; and (3) static measurement at fixed time points. None of these three premises holds for MetS drug validation.

For MetS, the therapeutically relevant endpoints are functional metabolic parameters—glucose uptake rate, lipid oxidation flux, insulin-stimulated phosphorylation of Akt and IRS-1, inflammatory cytokine secretion profiles, and cross-organ metabolic flux (e.g., hepatic glucose output vs. peripheral uptake)—none of which can be captured by cell viability or cytotoxicity assays alone (Wang et al., 2026).

The multi-tissue nature of MetS pathophysiology (encompassing adipose tissue, liver, skeletal muscle, pancreatic beta cells, gut microbiome, and hypothalamic signaling) demands co-culture or multi-organ-chip systems rather than single-tissue organoids. Current single-organoid models, however well differentiated, cannot recapitulate the inter-organ crosstalk that defines MetS progression (Juguilon et al., 2025).

The chronic, diet-induced, lifestyle-dependent progression of MetS requires dynamic, longitudinal, time-resolved measurement—a capability that most current organoid platforms, designed for acute perturbation experiments, rarely provide (Pushpa Ramesan et al., 2026). This mismatch is not a technical gap that can be closed by incremental improvement; it is a paradigm-level constraint. The solution requires establishing new evaluation standards centered on functional metabolic endpoints—a challenge that the priority research agenda (Section 7.2) addresses directly (Zhou et al., 2025).

### 6.4 AI-HTS and solid dispersion challenges

Beyond the validation platform challenges outlined above, recent advances in AI-driven high-throughput screening (Liu X. et al., 2026) have expanded the toolkit available, yet AI-driven TCM development faces a more fundamental pharmaceutical bottleneck: a substantial proportion of bioactive constituents in classic formulas—including flavonoids, terpenoids, and alkaloids—belong to BCS class II or IV, exhibiting extremely low aqueous solubility and limited oral bioavailability. Even when AI screening accurately identifies compounds or formula combinations with theoretical multi-target activity against MetS, the AI recommendation cannot translate into actual pharmacodynamic efficacy if these constituents fail to reach effective therapeutic concentrations *in vivo*. Solid dispersion technology, which disperses poorly soluble drugs in a hydrophilic carrier at the amorphous or molecular level, can markedly enhance dissolution rate and oral bioavailability. Machine learning has recently been introduced to the design and optimization of such formulation systems (Shen et al., 2026). Ma et al. employed nanocrystal-based solid dispersion to significantly improve andrographolide dissolution (Ma et al., 2018), achieving high drug loading and enhanced bioavailability. Xie et al. (Xie et al., 2019) prepared redispersible baicalin nanosuspensions using co-processed cellulose–sodium carboxymethyl starch as a carrier, achieving an approximately twofold improvement in oral bioavailability relative to the bulk drug. Such cases demonstrate that formulation technologies can deliver the bioavailability gains that AI-screened candidates require, although the studies themselves did not involve AI methods. Coupling solid dispersion technology with the organoid validation platforms discussed above would form an iterative loop of AI screening, solid dispersion formulation, metabolic organoid *in vitro* evaluation, and iterative optimization—a direction that merits priority attention. Each TCM formula, however, contains 6–12 medicinal materials, and solubilization–co-precipitation interactions among constituents complicate formulation design. This direction requires both AI-assisted formulation optimization algorithms and standardized, high-throughput *in vitro* dissolution–metabolism evaluation systems—both of which remain at an early stage (Shen et al., 2026; Semmelrock et al., 2025). A multi-dimensional comparison of traditional and AI-driven R&D paradigms is summarized in Table 3.

**TABLE 3:** Multi-dimensional comparison of traditional and AI-driven R&D paradigms for TCM formulas, juxtaposing the current actual state with the target state proposed in this review.

Dimension	Traditional paradigm	AI-driven (current actual state)	AI-driven (target state, proposed)
Screening method	Expert experience-driven, manual literature review	LLM generative reasoning + KG graph reasoning in research settings (P@30 = 35.62% (Wang et al., 2025b), not clinically validated)	Validated AI-assisted screening embedded in the R&D workflow (proposed)
Validation cycle	Months (animal experiments per formula)	Weeks in practice (multi-week for hepatic steatosis organoid culture (Liu et al., 2024a)); days-to-weeks demonstrated only on non-MetS platforms	Days-to-weeks validation with standardized functional metabolic endpoints (proposed)
Candidate throughput	Dozens per round	Thousands per round demonstrated on a rare-disease organoid platform (2,802 compounds in 13 days (Xu et al., 2025a)); not yet for TCM formulas	Thousands of candidates per round at the formula level (proposed)
Negative data feedback	Not recorded or published	Absent: no platform stores structured negative data (Gap 1); only isolated methodological proposals (Ivanov, 2026)	Structured storage of negative data with model reinjection (proposed)
Scalability	Linearly constrained by expert resources	Computational scaling of model iteration; experimental validation remains the bottleneck	Computational scaling including validation feedback loops (proposed)

### 6.5 Pharmacological evidence base of classic formulas for MetS

AI readiness claims for classic formula development must be anchored in the pharmacological evidence base they are intended to serve. For each formula discussed as a case in this review, six categories of information are required for pharmacological verifiability: the dose ranges tested, the minimum effective concentrations observed, the models used and their clinical relevance, the positive and negative controls applied, the duration of administration, and the type of extract (crude drug, fraction, or characterized preparation). This section assembles that evidence study by study, with *in vivo* models as the primary unit of appraisal and the paired *in vitro* systems reported by the same studies; candidate studies were identified through the structured search and citation tracing described in Section 2, and entries were compiled and verified against the primary reports. Characterization reporting follows the ConPhyMP checklist; the completed ConPhyMP Supplementary Tables 1, 2a for the case-formula medicinals are provided as a separate supplementary PDF.

The evidence is organized in a single evidence table with the following fields: formula and botanical composition; extract type and characterization; model (species, cell line, or organoid) and its relevance to MetS; dose range and minimum effective concentration; control settings; administration duration; measured outcomes (glycemic, lipid, inflammatory, and oxidative-stress endpoints); and source reference. The *in vivo* evidence, together with the paired *in vitro* systems used by the same studies, is consolidated in Table 4 (Xu P. et al., 2025; Lin et al., 2024; Liu R. et al., 2024; Zhang Y. et al., 2025; Zhang et al., 2012; Qian et al., 2016; Morita et al., 2022; Kawashima et al., 2019; Lin et al., 2014; Akaki et al., 2018; Guo et al., 2023; Chen et al., 2018; Zhang et al., 2021); clinical trial evidence is appraised separately in Section 6.6 (Table 5).

**TABLE 4:** *In-vivo* pharmacological evidence base for classic formulas and core constituents in MetS models (Xu P. et al., 2025; Lin et al., 2024; Liu R. et al., 2024; Zhang Y. et al., 2025; Zhang et al., 2012; Qian et al., 2016; Morita et al., 2022; Kawashima et al., 2019; Lin et al., 2014; Akaki et al., 2018; Guo et al., 2023; Chen et al., 2018; Zhang et al., 2021).

Formula/constituent	Model and relevance	Dose range; minimum effective dose	Controls	Duration	Extract and characterization
Huanglian Jiedu decoction (HLJDD) (Xu et al., 2025b)	C57BL/6J HFD obese mice; 3T3-L1 adipocytes	2.25 and 4.5 g/kg/d p.o.; 2.25 g/kg/d improved OGTT/ITT	Normal chow; HFD; 5%/10% drug serum + HDAC3 knockdown (*in vitro*)	4 weeks	Water extract; HDAC3-UCP1/PGC-1α mechanism; full HPLC profile not reported
HLJDD (Lin et al., 2024)	VSMC foam cells; ApoE mice	Drug-serum gradients	Model control	—	Water extract; P2RY12/autophagy (atherosclerotic component, supportive)
Gegen Qinlian decoction (GQD) (Liu et al., 2024b)	db/db mice (m/m controls)	Low/mid/high dose tiers; low tier improved glycemia	m/m blank; model; metformin positive	12 weeks	Water extract; metagenomics + targeted metabolomics (bile acid-TGR5)
GQD — puerarin-microbiota axis (Zhang et al., 2025b)	HFD/STZ and db/db mice	Puerarin; LBI 1 × 10^9^ CFU	Normal diet; model; FGF21-pathway validation	≥8 weeks	Puerarin (single compound); HDAC9-FGF21 mechanism
GQD dose-response (Zhang et al., 2012)	SD rats, obesity + low-dose STZ diabetic nephropathy	1.65–28.05 g crude drug/kg, 9 dose levels; effective window 8.25–14.85 g/kg	Normal; model; metformin	Per the published protocol	Water decoction; classical dose-response design [Chinese-language source]
Daisaikoto (Qian et al., 2016)	Wistar HFD/STZ diabetic fatty liver (n = 60)	Low/mid/high; high dose outperformed simvastatin	Control; HFD/STZ; simvastatin positive	8 weeks	Kampo granule; SIRT1/NF-κB protein and mRNA
Daisaikoto (Morita et al., 2022)	MC4R-deficient obese mice (n = 12/group)	10% drug-embedded high-fat diet	SD control; HFD control	4 weeks	Extract-embedded diet; metabolomics (hepatic TG degradation/FAO)
Daisaikoto (Kawashima et al., 2019)	C57BL/6 HFD-weight-cycling mice	Diet-embedded	Normal; HFD relapse control	Per the published protocol	Extract-embedded; 16S + metabolomics
Bofutsushosan (Lin et al., 2014)	C57BL/6J HFD 12-week mice	Three oral doses; all lowered TG/FFA/leptin (P < 0.01)	Normal chow; HFD + vehicle; rosiglitazone positive	4 weeks treatment after 12 weeks HFD	Water extract; AMPK/GLUT4
Bofutsushosan (Akaki et al., 2018)	ICR/Jcl HFD mice; Caco-2	Oral extract; Caco-2 gradient	HFD control	Fecal collection d6–23	Water extract + per-herb methanol extracts characterized individually
Baicalein (Guo et al., 2023)	C57BL/6 MCD-diet NAFLD mice	Diet-administered; lipid reduction and barrier repair	MCD model; normal diet	Throughout MCD period	Baicalein (single compound); ZO-1/claudin-1, SCFA
Puerarin (Chen et al., 2018)	SD HFD/STZ T2DM rats; L6/palmitate	Injection/gavage per protocol	Normal; model; μ-opioid receptor validation	Per the published protocol	Puerarin (single compound); mitochondrial biogenesis/FAO gene profile
Berberine (Zhang et al., 2021)	db/db diabetic nephropathy mice	100 and 200 mg/kg; 100 mg/kg lowered FBG, improved OGTT/ITT	db/m; model; metformin combination	8 weeks	Berberine (single compound); Trib1-dependent anti-inflammatory mechanism

**TABLE 5:** Clinical evidence for classic formulas and constituents in MetS and its components: trial-level appraisal (Azushima et al., 2015; Lee et al., 2014; Park et al., 2014; Liao et al., 2026; He H. et al., 2026; Jazayeri-Tehrani et al., 2019; Chan et al., 2022).

Trial/evidence	Design; sample	Intervention; duration	Key outcomes	Risk-of-bias/certainty notes
Bofutsushosan, obese hypertension (Azushima et al., 2015)	Multicenter, randomized, open-label; n = 107	Adjunct to antihypertensives vs. antihypertensives alone; 24 weeks	24-h ambulatory blood-pressure profile	Open-label, no placebo; moderate certainty
Bofutsushosan + probiotics (Lee et al., 2014)	Randomized, double-blind, placebo-controlled	BTS + probiotic vs. BTS + placebo capsule; 8 weeks	Body weight; metabolic endotoxemia; microbiota	Small sample; placebo covers probiotic arm only; moderate
Bofutsushosan pharmacogenomics (Park et al., 2014)	Randomized, double-blind, placebo-controlled + GNB3/ADRB3/UCP2/PPARγ subgroups	BTS vs. placebo; 12 weeks	BMI/body fat; genotype-dependent response	Small subgroups; Korean population; moderate
GQD for T2DM: meta-analysis (Liao et al., 2026)	Systematic review of RCTs; HOMA-IR pooled	GQD ± conventional vs. conventional; 8–12 weeks most effective	HOMA-IR, glycemia, HbA1c (P < 0.001)	High (pooled); constituent RCT quality heterogeneous
GQD + metformin (He et al., 2026b)	Systematic review of RCTs	GQD + metformin vs. metformin	T2DM efficacy and safety	Moderate-high; mostly single-comparator design
Nano-curcumin, NAFLD (Jazayeri-Tehrani et al., 2019)	Double-blind RCT; n = 84 (42/group)	2 × 40 mg/d nano-curcumin vs. placebo; 12 weeks	HDL, QUICKI, glycemic-lipid-inflammatory markers	Single center; Iranian population; moderate
Berberine, antipsychotic-induced MetS (Chan et al., 2022)	RCT (adjunctive design)	Add-on berberine vs. control	Weight gain; MetS components	Drug-induced MetS population; extrapolation cautioned; moderate

Throughout Sections 3–6, knowledge-graph edges lacking dose–time attributes (Gap 2) limit dose-aware reasoning, and validation-platform mismatches (Section 6.3) define which pharmacological claims can be tested at all. This section therefore consolidates the pharmacological literature underlying each bottleneck rather than restating those arguments. Pharmacological verifiability is applied as a binding constraint on technology readiness.

### 6.6 Clinical evidence status of MetS-related formulas

The clinical evidence for classic formulas in MetS and its components (central obesity, dyslipidemia, hypertension, hyperglycemia) is assessed here on three axes: risk of bias in randomized controlled trials, compliance with CONSORT-CHM reporting, and the certainty of evidence on a GRADE-style scale. This assessment anchors the AI-readiness argument in the evidence base it is meant to serve: a recommendation technology can only be as credible as the clinical evidence available for its training and validation.

Trial-level appraisal is provided in Table 5 (Azushima et al., 2015; Lee et al., 2014; Park et al., 2014; Liao et al., 2026; He H. et al., 2026; Jazayeri-Tehrani et al., 2019; Chan et al., 2022): for each trial or pooled analysis we tabulate the design and sample, intervention and duration, key outcomes, and a risk-of-bias/certainty note covering randomization, allocation concealment, blinding, selective reporting, and CONSORT-CHM items (formula composition disclosure, dosage form, batch quality control).

Across the reviewed trials, most evidence clusters at moderate certainty: blinding and allocation concealment are frequently under-reported, formula composition and batch quality are inconsistently documented per CONSORT-CHM, and effect sizes vary with population and genotype (Park et al., 2014). The two pooled analyses of Gegen Qinlian decoction reach high certainty for HOMA-IR improvement (Liao et al., 2026) and moderate-to-high certainty for add-on efficacy with metformin (He H. et al., 2026). These limitations propagate directly into the training data of any AI screening system (Section 5): a recommendation engine trained on trials with incomplete composition documentation inherits that opacity as irreducible label noise.

## 7 Bottlenecks and a priority research agenda

### 7.1 Structural bottlenecks

AI-driven transformation of TCM development faces bottlenecks across four dimensions (a framework we propose). At the knowledge–data level, the central problem is the absence of a negative space: existing TCM-KGs record only positive findings, without systematic accumulation of negative data. Semmelrock et al. have noted that machine learning research in general suffers from poor reproducibility (Semmelrock et al., 2025), and the subjectivity of TCM terminology and the ambiguity of syndrome definitions are likely to amplify this problem. In the MetS context, syndromes such as phlegm-dampness interior exuberance, qi–yin deficiency, and liver-depression spleen-deficiency depend on subjective consensus among TCM clinicians; the blurring of syndrome boundaries renders the definition of negative data substantially more challenging than in modern medicine. When the standard for efficacy varies by syndrome, the judgment of inefficacy loses a unified basis. The negative data generation method proposed by Ivanov has currently been validated only at the molecular docking level (Ivanov, 2026); whether it can be extended to TCM compound formulas remains unknown. At the model level, LLM hallucination in TCM contexts is further amplified by the multi-syndrome characteristics of MetS. Domain-specific fine-tuning may counterintuitively increase hallucination rates: GPT-4o achieved an F1 of only 0.625 on the high-difficulty category of the MedHallu benchmark (Pandit et al., 2025). In MetS, the boundaries among qi–yin deficiency, phlegm-dampness exuberance, and liver-depression spleen-deficiency are not entirely distinct, and LLM misclassification of syndromes would deprive subsequent formula recommendations of their diagnostic foundation. At the validation level, the lag is equally apparent: no consensus exists on efficacy endpoints for multi-component formula assessment (Li et al., 2025f); metabolic organoid culture cycles are prolonged and detection throughput limited; and translational data linking *in vitro* outcomes to clinical efficacy are scarce. The multi-dimensionality of functional metabolic endpoints in MetS means that improvement in any single indicator is insufficient to deem a formula effective, and no standardized evaluation system covering multiple metabolic dimensions simultaneously has yet been established. At the regulatory level, the situation is essentially a blank slate: no regulatory document specifies what evidentiary standards an AI-recommended formula must meet before entering clinical trials. Lee et al. have noted, from an FDA perspective, that a causal inference AI framework for determining therapeutic effectiveness and supporting regulatory decisions has yet to be developed; their causal graph model, by distinguishing correlation from causation, provides a methodological foundation for the regulatory evaluation of multi-target TCM formula effects (Lee et al., 2026).

We propose that “negative data” in TCM research be further discriminated into six states, because they carry different evidential weight and demand different handling: (1) genuinely negative results, i.e., adequately powered studies demonstrating absence of effect; (2) unvalidated absences, where no test has been performed; (3) publication-bias-induced absences, where null findings exist but were never reported; (4) assay-scope limitations, where the endpoint fell outside the assay’s sensitive range; (5) dose-range misses, where the tested window excluded the effective concentration; and (6) model-mismatch cases, where the biological model did not capture the relevant human pathomechanism. Only states (1) and (3) warrant negative edge weighting in a TCM-KG; states (4)–(6) should instead annotate the corresponding positive edges as unconfirmed in that context, whereas state (2), where no test has been performed, leaves the graph silent: absence of a record is not itself evidence and must not be encoded as a negative edge. Conflating these states inflates uncertainty in KG edge weights and, through the evidence-mapping rules of Section 2, biases component-level TRL assignments in Table 2. For machine readability, these six states are labeled tested-and-inactive, technically inconclusive, absent-from-database, never-studied, context-dependent activity, and contradicted findings, respectively; each state carries a typed encoding, and safeguards against false negatives entering downstream learning include minimum-evidence requirements, contradiction flagging with provenance, and a review workflow before an edge is written. The repository design is grounded in FAIR principles, with typed provenance on every edge (literature, case record, or experiment, each with an evidence grade).

### 7.2 From bottlenecks to breakthroughs: a priority research agenda

The four bottlenecks identified above map, in the authors’ proposal, onto four priority initiatives, each with a different implementation baseline. The first two are foundational. Structured publication and sharing of negative data: Ivanov has provided a methodological framework, yet methodology cannot substitute for institutional change; whether journals establish dedicated negative-result columns and whether conferences accept null-validation reports ultimately depend on collective editorial board decisions. The TCM-Hallu benchmark for hallucination evaluation is equally urgent: terminology ambiguity, compatibility contraindications, syndrome drift, and formula provenance issues require independent annotation by clinical TCM experts. The remaining two initiatives carry higher barriers to entry. The core of a minimum standardized MetS evaluation indicator set is the functional metabolic endpoint, which must span at minimum the glycemic, lipid, and inflammatory dimensions; high-throughput metabolic organoid culture represents the most significant engineering bottleneck, yet also the most tractable target for industry-wide consensus. The regulatory framework for AI-recommended formulas poses the core challenge for regulators: the NMPA category 3.1 pathway streamlines registration on the basis of traditional use (National Medical Products Administration, 2020), whereas AI-recommended formulas require a fundamentally different evidentiary standard, creating structural tension between the two. The causal AI framework advanced by Lee et al. provides a referenceable starting point (Lee et al., 2026). These four initiatives are not parallel in priority. The first two close the negative space—without closing it, the algorithms and standards comprising the subsequent two initiatives would operate on an incomplete foundation. This sequential logic mirrors the technical asymmetry revealed by the TRL grading: completing the knowledge infrastructure at the KG level is a prerequisite for advancing LLM algorithms, and evaluation standardization is, in turn, the evidentiary basis for regulatory frameworks. Reversing this order—for instance, developing more sophisticated LLMs before supplementing the knowledge base—would leave the reliability of higher-level technologies constructed on an unverifiable foundation.

Priority Research Agenda:

1. Institutionalize negative data collection: Establish a shared repository for failed observations (Sansom et al., 2024): clinical observations, absent therapeutic effects, and incompatible herb combinations—the structural antidote to KG reasoning bias.
2. Build the TCM-Hallu hallucination benchmark: Develop a dedicated evaluation dataset covering all three TCM hallucination categories, annotated by clinical TCM experts, to create an objective measurability baseline.
3. Establish multi-dimensional evaluation systems centered on functional metabolic endpoints: Develop new organoid differentiation protocols, multi-organ-chip architectures, and assay panels for functional metabolic readouts—moving beyond tumor PDO paradigms toward MetS-specific validation platforms.
4. Develop AI-assisted regulatory review frameworks: Create regulatory science frameworks that incorporate AI-generated evidence as supplementary data for TCM new drug evaluation.

## 8 Conclusion

This review has systematically examined the role and limitations of knowledge graphs, large language models, and high-throughput experimental technologies in transforming TCM development, using metabolic syndrome as a translational case study. Three findings constitute the core contributions of this work.

First, the three technology streams exhibit markedly asymmetric readiness. At the stream level, knowledge-graph approaches span TRL 3–6, large language model approaches span TRL 1–5, and high-throughput validation platforms span TRL 3–5 (component-level ratings in Table 2). The generative capability of the LLM stream has outpaced its evaluation infrastructure: no dedicated TCM hallucination evaluation benchmark yet exists. KG entity recognition is the most mature component, yet reasoning capabilities are constrained by three structural gaps: the absence of negative data, missing dose–time dimensions, and unencoded monarch–minister–assistant–envoy hierarchies. High-throughput experimentation is technically feasible but suffers from a systematic validation platform mismatch with MetS functional metabolic endpoints.

Second, tumor-derived organoid validation paradigms show limited direct transferability to metabolic disease. Tumor PDOs are built on three convergent premises—proliferation/death endpoints, single-tissue models, and static measurements—none of which applies to MetS. Functional metabolic endpoints, multi-tissue dynamic assessment, and chronic progression modeling require an entirely new evaluation framework. Partial precedents nevertheless exist: pluripotent stem cell-derived human liver organoids with functional metabolic readouts have been established for steatohepatitis (Ouchi et al., 2019), indicating adaptation paths rather than a categorical barrier.

Third, the precision phytomedicine transformation must follow a “foundations before algorithms” sequence. Until the “negative space” in the knowledge infrastructure—negative data—is filled, algorithm-level advances will operate in an incomplete information space. The actionable priority agenda is: institutionalize negative data collection, build the TCM-Hallu hallucination benchmark, establish multi-dimensional evaluation systems centered on functional metabolic endpoints, and develop AI-assisted regulatory review frameworks.

Traditional Chinese medicine is not short of formulas; it is short of reliable methods to screen and validate those formulas. The fundamental task of AI-driven transformation is not to pursue algorithmic precision but to fill the structural gaps in the knowledge infrastructure that make algorithmic advances meaningful.

For the Chinese medicine research community, this review underscores an urgent imperative: the computational advances of AI must be anchored in the faithful digitization of indigenous knowledge systems. Only by treating classic formulas not merely as data sources but as curated traditional medicine artifacts can the field ensure that algorithmic innovation serves rather than erodes the accumulated medical heritage of traditional cultures.

A final point concerns reproducibility. Based on the evidence standards developed in this review, a published AI-based study of classic formulas should be regarded as non-reproducible, and its claims unverifiable, whenever any of the following core standards is unmet. First, the herbal material must be fully characterized. The six provenance dimensions proposed in Section 3.3 specify the minimum attributes: validated botanical names, plant part, processing (paozhi) status, extraction method and dosage form, herb proportions, and the source text variant. Second, the pharmacological test parameters must be reported in sufficient detail to allow verification. At minimum, studies should state the dose range tested, the minimum effective concentration observed, the model system and its clinical relevance, the positive and negative controls, the treatment duration, and the type of extract, as tabulated in Section 6.5. Third, any readiness claim should state its rating unit and intended use explicitly and should be supported by evidence of prospective utility, independent replication, and comparison against non-AI baselines (Section 2; Supplementary Table S1). Fourth, negative results should be archived, and the evaluation benchmark, once established, should itself be openly available, so that reasoning steps can be examined rather than accepted on trust. Computational methods can support the enforcement of these standards, for example, through schema-level validation that flags missing attributes, benchmark-based evaluation of generative outputs, and structured repositories for negative data. They cannot, however, replace the experiments and clinical verification that generate the underlying evidence. Only studies that satisfy these core quality standards should be described as ready to use; all others remain at the hypothesis-generating stage.

This review was additionally checked against the four pillars of ethnopharmacological research—local (indigenous) knowledge dynamics, substantiation, reflexivity, and health equity. Local knowledge dynamics: Section 3.3 and the Conclusion treat classic formula texts as curated indigenous knowledge artifacts whose digitization must preserve jun-chen-zuo-shi context rather than flatten it into generic triples. Substantiation: Sections 5 and 6 anchor readiness claims in experimentally or clinically verifiable evidence, and Section 6.5 tabulates the pharmacological evidence base formula by formula. Reflexivity: Sections 2 and 7 explicitly separate literature-supported conclusions from the authors’ own proposals, and flag the latter as such. Health equity: Section 6.6 addresses the quality and accessibility of clinical evidence, and the Conclusion links AI-assisted screening to affordable MetS management. The consensus-based methodology for substantiating indigenous medicinal plant use established in ethnopharmacology (Heinrich et al., 1998) informs this framing.
