# Supplementary material for: Artificial intelligence for Traditional Chinese Medicine classic formula development: a technology readiness assessment using metabolic syndrome as a case study
Source: Front Pharmacol. 2026 Oct 28;17:1970742. doi: 10.3389/fphar.2026.1970742 (PMC13752572; doi:10.3389/fphar.2026.1970742)
Supplement: Supplementary file 1 [file DataSheet1.pdf]

# ConPhyMP Tables 1 and 2a

**Manuscript:** Artificial Intelligence for Traditional Chinese Medicine Classic Formula Development: A Technology Readiness Assessment (Front. Pharmacol., Sec. Ethnopharmacology; MS No. 1970742)

**Guideline:** Heinrich M., Jalil B., Abdel-Tawab M., et al. Best Practice in the chemical characterisation of extracts used in pharmacological and toxicological research — The ConPhyMP — Guidelines. Front. Pharmacol. 13:953205 (2022). doi: 10.3389/fphar.2022.953205

Scope statement

**Note on table structure: the tables below follow the item structure of the ConPhyMP checklist as published — its first checklist table ("reporting of the starting plant material and its initial processing") constitutes Table 1, and its second ("most suitable analytical methods for defining the chemical profile") constitutes Table 2a, as requested by the journal. Item numbering follows the published checklist (Heinrich et al., 2022); the structure was verified against the published guideline article (https://www.frontiersin.org/journals/pharmacology/articles/10.3389/fphar.2022.953205).**

## TABLE 1. Reporting of the starting plant material and its initial processing

| | | |
|---|---|---|
| **Title and abstract** | **1(a) Title includes plant material, approach, therapeutic focus** | **Review title covers AI approach and TCM classic-formula development with MetS focus; individual plant names are not the study object (they appear in the reviewed literature).** |
| | 1(b) Informative, balanced abstract | Provided in manuscript Abstract. |
| Description of the botanical drug and taxonomic authentication | 2(a) Fully authenticated species names checked against MPNS / POWO / WFO | The four representative crude drugs discussed for the MetS case study are all Chinese Pharmacopoeia (ChP 2020) monographed species; accepted names verified against World Flora Online (see .md): *Astragalus mongholicus* Bunge (syn. *A. membranaceus* Fisch. ex Bunge), Fabaceae; *Salvia miltiorrhiza* Bunge, Lamiaceae; *Pueraria lobata* (Willd.) Ohwi (syn. *P. montana* var. *lobata*), Fabaceae; *Coptis chinensis* Franch., Ranunculaceae. |
| | 2(b) Pharmacopoeial names checked against taxonomic databases | Done — see companion botanical nomenclature table; each entry gives WFO ID and verification status. |
| | 2(c) Locality, date, season of collection | Not applicable — literature-based review; no material collected by the authors. |
| | 2(d) Voucher specimen deposition | Not applicable — literature-based review; no material collected by the authors. |
| | 2(e) Authentication method and personnel | Not applicable — literature-based review. |
| | 2(f) Special observations on collected samples | Not applicable — literature-based review. |
| Description of the extract and extraction process | 3(a) Full species names incl. authority and family; pharmacopoeial drug name | Species names with authorities and families given above; pharmacopoeial drug names (ChP): Huangqi (Astragali Radix), Danshen (Salviae Miltiorrhizae Radix et Rhizoma), Gegen (Puerariae Lobatae Radix), Huanglian (Coptidis Rhizoma). Wording verified against the ChP 2020 monograph nomenclature on 2026-09-29. |
| | 3(b) Plant parts used | Huangqi: root; Danshen: root and rhizome; Gegen: root; Huanglian: rhizome (per ChP monograph definitions). |
| | 3(c) Drug–solvent ratio, drug–extract ratio | Not applicable — literature-based review; no extract prepared by the authors. |

| | | |
|---|---|---|
| | 3(d) Pre-extraction/fermentation procedures and storage | Not applicable — literature-based review. |
| | 3(e) Extraction solvent | Not applicable — literature-based review. |
| | 3(f) Type of extract by consistency | Not applicable — literature-based review. |
| | 3(g) Mode of extraction | Not applicable — literature-based review. |
| | 3(h) Pressure, temperature, duration | Not applicable — literature-based review. |
| | 3(i) Drying mode | Not applicable — literature-based review. |
| | 3(j) Traditional processing (paozhi) | The reviewed literature on classic formulae describes traditional decoction of the crude drugs; no processing was performed by the authors. Not applicable — literature-based review. |
| | 3(k) Other processing/purification steps | Not applicable — literature-based review. |
| | 3(l) Ratios of individual botanical drugs in multicomponent extracts | Not applicable — literature-based review; formula compositions cited from the reviewed publications. |
| | 3(n) Content of active ingredients / markers per pharmacopoeial monograph | Not applicable — literature-based review; the manuscript does not report quantitative content data. |
| | 3(o) Added antimicrobials / preservatives | Not applicable. |
| | 3(p) Added excipients | Not applicable. |
| Legal basis for collection and processing | 4(a) Nagoya protocol / CITES compliance | Not applicable — no material collected; only published literature is analysed. |
| | 4(b) Phytosanitary compliance | Not applicable. |
| | 4(c) Collaboration with source-country institutions | Not applicable; no field collection. |
| Finished (commercial) product characteristics | 5(a–i) Product name, manufacturer, pharmaceutical form, extract amount, ingredients, excipients, regulatory status, batch number, storage | Not applicable — literature-based review; no commercial product was used. |

## TABLE 2a. Analytical methods for defining the chemical profile (by extract type)

| **1. Type of extract** | **Declare A / B / C** | **Not applicable — literature-based review; no extract used.** |
|---|---|---|
| 2. Preferred/main methods: pharmacopoeial compliance; triple chemical fingerprinting; quantification of ≥2 marker compounds | Required for types A/B | |
| 3. Alternative methods (single fingerprinting with ≥3 detection parameters) | Alternative for A/B/C | Not applicable — literature-based review. |
| 4. Use of reference standards | Direct overlay with reference-standard chromatograms | Not applicable — literature-based review. |
| 5. Comparison of different extracts/samples of the same plant | Similarity scoring | Not applicable — literature-based review. |

**Companion botanical nomenclature verification:** see the species-by-species WFO verification table (accepted name, synonyms, family, plant part, pinyin) submitted alongside.

**Sources consulted:** ConPhyMP guideline full text (frontiersin.org/articles/10.3389/fphar.2022.953205/full, accessed 2026-09-28); World Flora Online name-matching service (accessed 2026-09-28, classification version 2026-06); Chinese Pharmacopoeia 2020 marker-compound designations (verified 2026-09-29).
