# Supplementary material for: Artificial intelligence for Traditional Chinese Medicine classic formula development: a technology readiness assessment using metabolic syndrome as a case study
Source: Front Pharmacol. 2026 Oct 28;17:1970742. doi: 10.3389/fphar.2026.1970742 (PMC13752572; doi:10.3389/fphar.2026.1970742)
Supplement: Supplementary file 2 [file Table1.docx]

**Supplementary Table S1.** Technology readiness scoring matrix (component level)

| **Component** | **Rating unit** | **Intended use** | **TRL** | **Software performance** | **Data/domain fit** | **Biological validity** | **External validation** | **Prospective utility** | **Independent replication** | **Non-AI baseline comparison** | **Evidence (refs)** | **Decision note** |
| --- | --- | --- | --- | --- | --- | --- | --- | --- | --- | --- | --- | --- |
| TCM NER | Component | Retrieval | TRL 4–5 | BERT F1 90.7% doc-level joint extraction [36]; BERT-BiLSTM-CRF staged [35] | TCM corpora; OOD classical texts/low-frequency entities unassessed | n/a | n/a | No (retrospective corpus recovery) | No | No head-to-head non-AI baseline reported | [36] | Domain-corpora validation only; clinical consequences of errors unquantified |
| KG construction | Component | Retrieval | TRL 5–6 | TCMKD, TCMBank deployed and publicly accessible [43,44] | Real TCM text/KG at research scale | n/a | n/a | No | Partial (independent use by third parties) | n/a | [43,44] | Research-grade deployment supports TRL 5–6 |
| GNN graph reasoning | Component | Candidate prioritization | TRL 3–4 | DREAMwalk +16.8% on general drug–disease pairs [100]; MRHormer inductive [102] | General pairs; TCM formulas untested | n/a | n/a | No | No | Outperforms state-of-the-art link prediction baselines with average accuracy 0.873 across three biomedical KGs (best walk-based baseline NEWMIN 0.840, best GNN WalkPool 0.760, best transition-based ComplEx 0.706); drug–disease association accuracy improved by up to 16.8% | [100,102] | Validated only on general drug–disease pairs, not TCM formulas |
| LLM formula recommendation | Component | Hypothesis generation | TRL 3 | P@30 35.62%, F1@30 37.36% (Wang et al., 2025) [85] | TCM corpus; MetS multi-syndrome untested | None (no functional validation) | None beyond original developers | No (retrospective benchmark only) | No | n/a | [85] | Retrospective retrieval performance does not evidence prospective utility; no clinical validation |
| Multi-agent orchestration | Component | Candidate prioritization | TRL 2–3 | AgentClinic MedQA 60.3%, HLE 8.6% [93] | General medicine; no TCM formula-safety orchestration | n/a | n/a | No | No | n/a | [92,93] | Rating extrapolated from general medical agents; no TCM/MetS coverage |
| GraphRAG (TCM) | Component | Hypothesis generation | TRL 1–2 | Concept stage [89] | Not yet implemented for TCM | n/a | n/a | No | No | n/a | [89] | Concept formulated; no experimental proof of concept |
| Metabolic organoid validation | Component | Regulatory evidence (preclinical) | TRL 3–4 | n/a (assay-type) | Hepatic steatosis organoid culture 4–8 weeks [106] | Functional endpoint; maturation time and throughput limited | n/a | n/a | n/a | No head-to-head non-AI baseline reported | [106] | Maturation time and throughput constraints noted in criteria |
| Organ-chip integration | Component | Regulatory evidence (preclinical) | TRL 3–4 | n/a (assay-type) | Intestine–liver dual-chip demonstrated [110] | Dual-tissue only; ≥3-tissue chip not reported as of June 2026 (authors' search) | n/a | n/a | n/a | No head-to-head non-AI baseline reported | [110] | No chip integrating more than three metabolically relevant tissues reported |
| AI-HTS screening | Component | Candidate prioritization | TRL 4–5 | AI-assisted HTS workflows reviewed (see Section 6.4) [118] | General compound libraries | Partial (in vitro readouts linked) | n/a | No | Partial | Not verifiable; claim removed | [118] | Outcomes described in defined terms rather than via a second-hand metric (Section 6.4) |

*Note: TRL levels follow the definitions in Section 2; levels 7–9 are not assigned in this review. Retrospective-recovery performance (e.g., P@30 on curated benchmarks) is explicitly not treated as evidence of prospective utility. Ratings were assigned by the author team through consensus assessment against the published criteria (Section 2); no inter-rater agreement statistic is claimed. Evidence numbers refer to the manuscript reference list. A concordance of the values as submitted and the values in the revised manuscript follows the matrix below.*

Supplementary Table S1 (continued). Concordance of technology readiness values: values as submitted → values in the revised manuscript, by location and rating unit.

| **Location** | **Rated object (rating unit)** | **Value as submitted** | **Value in the revised manuscript** |
| --- | --- | --- | --- |
| Abstract; Conclusions | Knowledge graphs (stream) | 5–6 | 3–6 |
| Abstract; Conclusions | Large language models (stream) | 4–5 | 1–5 |
| Abstract; Conclusions | High-throughput validation (stream) | 4–5 | 3–5 |
| Table 2 | TCM NER (component) | 6–7 | 4–5 |
| Table 2 | KG construction (component) | 5–6 | 5–6 |
| Table 2 | GNN graph reasoning (component) | 4–5 | 3–4 |
| Table 2 | LLM formula recommendation (component) | 2–3 | 3 |
| Table 2 | Multi-agent orchestration (component) | 3–4 | 2–3 |
| Table 2 | Metabolic organoid validation (component) | 3–4 | 3–4 |
| Table 2 | Organ-chip integration (component) | 3–4 | 3–4 |
| Table 2 | AI-HTS screening (component) | 4–5 | 4–5 |
| Section 5.3 | GraphRAG in the TCM domain (component) | 1–2 | 1–2 |
| Figure 1 | Knowledge formalization (STAGE 1) | 5–6 | 4–6 |
| Figure 1 | Intelligent screening (STAGE 2) | 2–4 | 2–4 |
| Figure 1 | Closed-loop validation (STAGE 3) | 3–5 | 3–5 |
| Figure 2 (three-layer pipeline) | Output candidate ranking (output box) | 2–3 | 3 |
| Figure 3 (complementary architecture) | KG stream; LLM stream | 5–6; 4–5 | 3–6; 1–5 |
| Graphical abstract | KG stream; LLM stream; high-throughput | 5–6; 2–5; 4–5 | 3–6; 1–5; 3–5 |
