# Supplementary material for: Artificial intelligence for Traditional Chinese Medicine classic formula development: a technology readiness assessment using metabolic syndrome as a case study
Source: Front Pharmacol. 2026 Oct 28;17:1970742. doi: 10.3389/fphar.2026.1970742 (PMC13752572; doi:10.3389/fphar.2026.1970742)
Supplement: Supplementary file 3 [file Table2.docx]

# Supplementary Table S2. Coverage of the primary MetS-related TCM syndrome patterns in publicly available TCM knowledge platforms

| **Syndrome pattern** | **Synonyms queried** | **SymMap** | **TCMSP** | **TCMBank** | **HIT** | **TCMID** | **TCMKD** |
| --- | --- | --- | --- | --- | --- | --- | --- |
| Phlegm-dampness interior exuberance | 痰湿内蕴；痰湿中阻；痰湿阻滞 | Not retrieved | No syndrome entity class | No syndrome entity class | No syndrome entity class (per published scope) | No syndrome entity class (per published scope) | Not retrieved |
| Dampness-heat accumulation | 湿热内蕴；湿热蕴结；湿热中阻 | Retrieved (as 湿热中阻) | No syndrome entity class | No syndrome entity class | No syndrome entity class (per published scope) | No syndrome entity class (per published scope) | Retrieved (as TCMSY051 湿热中阻证; TCMSY235 湿热蕴结证) |
| Liver-depression with spleen-deficiency | 肝郁脾虚；肝郁脾虚证 | Not retrieved | No syndrome entity class | No syndrome entity class | No syndrome entity class (per published scope) | No syndrome entity class (per published scope) | Retrieved (as TCMSY243 肝郁脾虚证) |
| Qi-yin deficiency | 气阴两虚；气阴亏虚 | Retrieved | No syndrome entity class | No syndrome entity class | No syndrome entity class (per published scope) | No syndrome entity class (per published scope) | Retrieved (as TCMSY042 气阴两虚证; TCMSY043 气阴亏虚证) |
| Qi deficiency | 气虚；气虚证 | Retrieved | No syndrome entity class | No syndrome entity class | No syndrome entity class (per published scope) | No syndrome entity class (per published scope) | Retrieved (as TCMSY035 气虚证) |
| Yin deficiency | 阴虚；阴虚证 | Retrieved | No syndrome entity class | No syndrome entity class | No syndrome entity class (per published scope) | No syndrome entity class (per published scope) | Retrieved (as TCMSY176 阴虚证) |
| Blood stasis | 血瘀；血瘀证；气虚血瘀 | Retrieved | No syndrome entity class | No syndrome entity class | No syndrome entity class (per published scope) | No syndrome entity class (per published scope) | Retrieved (as TCMSY158 血瘀证; TCMSY229 气虚血瘀证) |
| Hyperactivity of liver yang | 肝阳上亢；肝阳上亢证 | Not retrieved | No syndrome entity class | No syndrome entity class | No syndrome entity class (per published scope) | No syndrome entity class (per published scope) | Not retrieved |
| Spleen deficiency with dampness retention | 脾虚湿蕴；脾虚湿盛；脾虚湿困 | Not retrieved | No syndrome entity class | No syndrome entity class | No syndrome entity class (per published scope) | No syndrome entity class (per published scope) | Not retrieved |
| Deficiency of both kidney yin and yang | 肾阴阳两虚；阴阳两虚 | Not retrieved | No syndrome entity class | No syndrome entity class | No syndrome entity class (per published scope) | No syndrome entity class (per published scope) | Retrieved (as TCMSY226 阴阳两虚证) |
| **Coverage** | **—** | **50% (5/10)** | **0% (0/10)** | **0% (0/10)** | **0% (0/10)** | **0% (0/10)** | **70% (7/10)** |

Note: Reference patterns were taken from two real-world MetS cohorts: Axinbai et al. (2024; n = 695; the 20 patterns reported at >1% frequency and the six basic patterns from latent structure analysis) and Xia et al. (2020; n = 1,262; 18 syndrome elements, with phlegm and dampness the most frequent). Each pattern was queried against the syndrome index or entity listings of each platform: SymMap, TCMSP and TCMBank on 29 September 2026, and TCMKD on 1 October 2026 at its new host (cbcb.cdutcm.edu.cn) after the original domain (tcmkd.com) ceased resolving; the interfaces of HIT and TCMID were unreachable on both dates. For SymMap, whose syndrome index is structured (233 entries derived from the Chinese Pharmacopoeia 2015 and 2020), the downloaded index was searched directly, and the TCMKD syndrome index (262 entries) was searched with the same protocol. A pattern was counted as covered for a platform if an entry with the same name or a documented synonymous name was present; the Coverage row reports synonym-inclusive rates. Mean coverage across the six platforms was 20.0% on this criterion (SymMap 5/10, TCMKD 7/10) and 15.0% using exact-name matching only (SymMap 4/10, TCMKD 5/10). For TCMSP and TCMBank, the platforms’ own entity listings contain no TCM syndrome entity class. HIT 2.0 hosts herbal-ingredient-target records only (Yan D, et al. Nucleic Acids Research 2022;50:D1238–D1243) and TCMID 2.0 encompasses prescriptions, herbs, ingredients, diseases, drugs and targets without a syndrome entity class (Huang L, et al. Nucleic Acids Research 2018;46:D1117–D1120); these four platforms are therefore scored 0% for syndrome coverage per their published data scopes.
