# Supplementary material for: Artificial intelligence for Traditional Chinese Medicine classic formula development: a technology readiness assessment using metabolic syndrome as a case study
Source: Front Pharmacol. 2026 Oct 28;17:1970742. doi: 10.3389/fphar.2026.1970742 (PMC13752572; doi:10.3389/fphar.2026.1970742)
Supplement: Supplementary file 4 [file Table3.docx]

# Supplementary Table S3. Search documentation for the narrative review

| Database | Search string | Window | Records | Executed |
| --- | --- | --- | --- | --- |
| PubMed (primary) | ("artificial intelligence"[tiab] OR "machine learning"[tiab] OR "deep learning"[tiab] OR "knowledge graph"[tiab] OR "large language model"[tiab]) AND ("traditional Chinese medicine"[tiab] OR "Chinese medicine"[tiab] OR "Chinese herbal"[tiab] OR "classic formula"[tiab] OR "classical formula"[tiab] OR TCM[tiab]) | 2007/01/01–2026/06/30 | 1,221 | 2026-09-29 |
| PubMed (MetS-restricted) | Primary strategy AND ("metabolic syndrome"[tiab] OR "metabolic syndromes"[tiab] OR obesity[tiab] OR diabetes[tiab] OR "insulin resistance"[tiab]) | 2007/01/01–2026/06/30 | 77 | 2026-09-29 |
| PubMed (technology-extended) | (Primary AI group OR "graph neural network"[tiab] OR organoid[tiab] OR "organ-on-a-chip"[tiab]) AND TCM group | 2007/01/01–2026/06/30 | 1,284 | 2026-09-29 |
| Web of Science Core Collection | TS=("artificial intelligence" OR "machine learning" OR "deep learning" OR "knowledge graph" OR "large language model") AND TS=("traditional Chinese medicine" OR "Chinese medicine" OR "Chinese herbal" OR "classic formula" OR "classical formula" OR TCM) (Core Collection edition) | 2007-01-01–2026-06-30 | 1,522 | 2026-09-30 |
| CNKI | SU=('人工智能'+'机器学习'+'深度学习'+'知识图谱'+'大语言模型') and SU=('中医药'+'中药'+'经典名方') (professional search, journal-article database) | 2007–2026 (year-level window) | 1,934 | 2026-09-30 |
| Wanfang Data | 主题:("人工智能" OR "机器学习" OR "深度学习" OR "知识图谱" OR "大语言模型") AND 主题:("中医药" OR "中药" OR "经典名方") (Smart Search, all resource types) | 2007–2026 (year-level window) | 5,101 | 2026-09-30 |

Note: Window rationale: the window opens in January 2007, when Hopkins introduced the concept of network pharmacology (manuscript reference [10]). Records were selected for relevance to the three technology streams (knowledge graphs, large language models, high-throughput validation) and the metabolic-syndrome case study, with citation tracing of key reviews; the 149 cited works constitute the final evidence base. The article is a critical narrative review; no PRISMA flow diagram applies. Quantitative record counts are reported for all four databases: the three PubMed strategies were executed on 2026-09-29, and the Web of Science Core Collection, CNKI, and Wanfang searches were executed on 2026-09-30 during the revision; all counts are exact database outputs, not estimates. Scope notes: the Web of Science search used the Core Collection edition with a custom publication-date window (2007-01-01 to 2026-06-30); a parallel run of the same strategy across All Databases returned 2,542 records. The CNKI search was run in the journal-article database with professional-search syntax; its interface supports year-level windows only, so the window was set to 2007-2026 (extending the upper bound to the full 2026 calendar year relative to the PubMed window). The Wanfang search used the documented subject query in Smart Search across resource types with a year-level window of 2007-2026; its journal-article subset comprises 3,336 records.
