# Supplementary material for: Artificial intelligence for Traditional Chinese Medicine classic formula development: a technology readiness assessment using metabolic syndrome as a case study
Source: Front Pharmacol. 2026 Oct 28;17:1970742. doi: 10.3389/fphar.2026.1970742 (PMC13752572; doi:10.3389/fphar.2026.1970742)
Supplement: Supplementary file 5 [file Table4.docx]

**Supplementary Table S4.** Botanical nomenclature verification

Manuscript: Artificial Intelligence for Traditional Chinese Medicine Classic Formula Development (manuscript no. 1970742). Nomenclature verified against World Flora Online (WFO; matching service, accessed 28 September 2026) and cross-checked against the Kew Royal Botanic Gardens name backbone (Plants of the World Online, accessed 30 September 2026), which underlies the Medicinal Plant Names Services (MPNS) scientific-name index.

| **Chinese name (pinyin)** | **Name used in the manuscript** | **Synonymy and source treatment** | **Family** | **Medicinal part** | **Verification record** |  |  |
| --- | --- | --- | --- | --- | --- | --- | --- |
| Huangqi (黄芪, Huángqí) | Astragalus mongholicus Bunge | Syn.: Astragalus membranaceus Fisch. ex Bunge. WFO places A. membranaceus under A. mongholicus; Kew POWO likewise lists A. mongholicus as accepted (IPNI lsid 478720-1) and A. membranaceus as its synonym (lsid 478611-1). The two-source treatment agrees with the manuscript usage (A. mongholicus, with A. membranaceus noted as synonym). | Fabaceae | Root | WFO wfo-0000185128 (accepted); POWO 478720-1 (accepted); A. membranaceus = POWO 478611-1 (synonym). Verified 28 and 30 September 2026. |  |  |
| Danshen (丹参, Dānshēn) | Salvia miltiorrhiza Bunge | No frequently used synonyms; WFO returns no candidate synonym records. Accepted name in both sources. | Lamiaceae | Root and rhizome | WFO wfo-0000301608 (accepted). Verified 28 September 2026. |  |  |
| Gegen (葛根, Gégēn) | Pueraria lobata (Willd.) Ohwi | Two coexisting taxonomic treatments: species-level P. lobata (Willd.) Ohwi (followed by the Chinese Pharmacopoeia [ChP 2020] and mainstream pharmacognosy literature) versus variety-level Pueraria montana var. lobata (Willd.) Maesen & S.M.Almeida ex Sanjappa & Predeep (the accepted name in the Kew backbone; several allied species are listed as its synonyms). Both names have independent WFO entries. The manuscript follows the ChP 2020 species-level usage; the Kew variety-level treatment is noted here for transparency. | Fabaceae | Root | WFO wfo-0000182939 (P. lobata); wfo-0000193854 (P. montana var. lobata). Kew POWO lists var. lobata as accepted. Verified 28 and 30 September 2026. |  |  |
| Huanglian (黄连, Huánglián) | Coptis chinensis Franch. | Pharmacopoeial source species. Closely related C. deltoidea C.Y.Cheng et Hsiao and C. teeta Wall. are also traded as Huanglian, but ChP 2020 primarily specifies C. chinensis. | Ranunculaceae | Rhizome | WFO wfo-0000619848 (accepted). Verified 28 September 2026. |  |  |

Note: Four botanical Latin names appear in the revised manuscript: Astragalus mongholicus Bunge and Salvia miltiorrhiza Bunge in the Introduction (references [4] and [3], respectively), and Pueraria lobata (Willd.) Ohwi and Coptis chinensis Franch. in the Introduction and the pharmacological evidence section (Sections 6.5–6.6); all other herbal drugs are referred to by their pinyin names (Huangqi, Danshen, Gegen, Huanglian), used consistently throughout. The four representative herbs of the case formulas are all included in the Chinese Pharmacopoeia (ChP 2020 edition). The reference set of the manuscript therefore contains no unresolved nomenclature inconsistency: the single nomenclatural update applied during revision (A. membranaceus to A. mongholicus, per reference [4] and both verification sources) is now used throughout.
